# HMGA1 acts as an epigenetic gatekeeper of ASCL2 and Wnt signaling during colon tumorigenesis

**DOI:** 10.1172/JCI184442

**Published:** 2025-02-03

**Authors:** Li Z. Luo, Jung-Hyun Kim, Iliana Herrera, Shaoguang Wu, Xinqun Wu, Seong-Sik Park, Juyoung Cho, Leslie Cope, Lingling Xian, Bailey E. West, Julian Calderon-Espinosa, Joseph Kim, Zanshé Thompson, Isha Maloo, Tatianna Larman, Karen L. Reddy, Ying Feng, Eric R. Fearon, Cynthia L. Sears, Linda Resar

**Affiliations:** 1 Division of Hematology, Department of Medicine, the Johns Hopkins University School of Medicine, Baltimore, Maryland, USA.; 2 Research Institute, National Cancer Center, Goyang-si, Gyeonggido, Republic of Korea.; 3 Division of Infectious Diseases, Department of Medicine,; 4 Sidney Kimmel Comprehensive Cancer Center, Division of Biostatistics,; 5 Pathobiology Graduate Program, Department of Pathology, and; 6 Human Genetics Graduate Program, Department of Genetics and Molecular Medicine, the Johns Hopkins University School of Medicine, Baltimore, Maryland, USA.; 7 Biochemistry and Molecular Biology Program, Johns Hopkins Bloomberg School of Public Health, Baltimore, Maryland, USA.; 8 Department of Pathology and; 9 Department of Biological Chemistry, the Johns Hopkins University School of Medicine, Baltimore, Maryland, USA.; 10 Department of Oncology, University of Michigan, Ann Arbor, Michigan, USA.; 11 Department of Oncology, Sidney Kimmel Comprehensive Cancer Center, and; 12 Molecular Immunology, the Johns Hopkins University School of Medicine, Baltimore, Maryland, USA.

**Keywords:** Oncology, Colorectal cancer, Epigenetics, Transcription

## Abstract

Mutated tumor cells undergo changes in chromatin accessibility and gene expression, resulting in aberrant proliferation and differentiation, although how this occurs is unclear. HMGA1 chromatin regulators are abundant in stem cells and oncogenic in diverse tissues; however, their role in colon tumorigenesis is only beginning to emerge. Here, we uncover a previously unknown epigenetic program whereby HMGA1 amplifies Wnt signaling during colon tumorigenesis driven by inflammatory microbiota and/or *Adenomatous polyposis coli* (*Apc*) inactivation. Mechanistically, HMGA1 “opens” chromatin to upregulate the stem cell regulator, *Ascl2,* and downstream Wnt effectors, promoting stem and Paneth-like cell states while depleting differentiated enterocytes. Loss of just one *Hmga1* allele within colon epithelium restrains tumorigenesis and Wnt signaling driven by mutant *Apc* and inflammatory microbiota. However, HMGA1 deficiency has minimal effects in colon epithelium under homeostatic conditions. In human colon cancer cells, *HMGA1* directly induces *ASCL2* by recruiting activating histone marks. Silencing *HMGA1* disrupts oncogenic properties, whereas reexpression of *ASCL2* partially rescues these phenotypes. Further, *HMGA1* and *ASCL2* are coexpressed and upregulated in human colorectal cancer. Together, our results establish HMGA1 as an epigenetic gatekeeper of Wnt signals and cell state under conditions of *APC* inactivation*,* illuminating HMGA1 as a potential therapeutic target in colon cancer.

## Abstract 2

HMGA1 promotes colon tumorigenesis by opening chromatin and amplifying Wnt signaling to maintain a stem-like state, highlighting HMGA1 as a promising potential therapeutic target.

## Graphical abstract

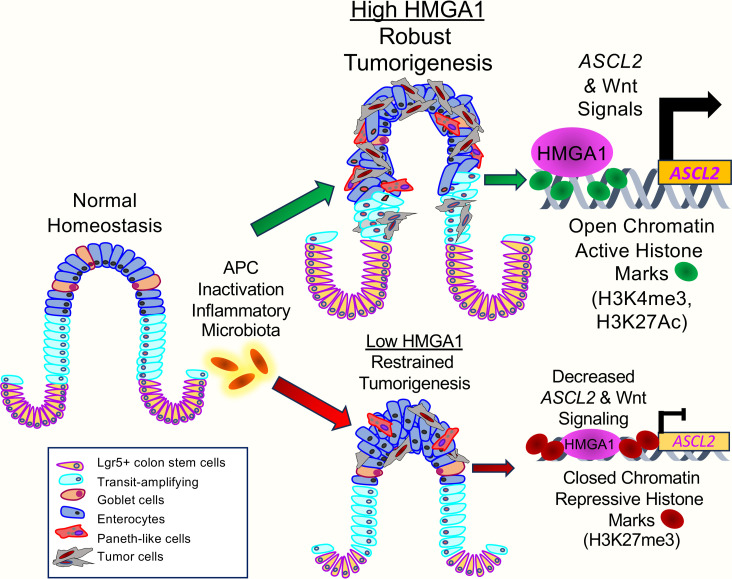

## Introduction

Exquisite control of transcriptional networks that regulate plasticity and other stem cell properties allow for tissue specification during embryogenesis and tissue maintenance after birth (1–9). Often referred to as “stemness” networks, genes involved in self-renewal and plasticity are largely silenced in differentiated tissues, although a subset remain active in adult stem cells where they contribute to tissue regeneration during homeostatic conditions or following injury and other stressors (3–6). By contrast, neoplastic cells frequently corrupt these same transcriptional networks to foster aberrant growth and differentiation (1–7, 9, 10). Moreover, tumor progression is associated with increased expression of genes controlling stem cell properties, which may foster the emergence of highly plastic tumor cells capable of metastatic progression, immune evasion, and therapy resistance (1–7, 9, 10). While mechanisms responsible for cell state during tissue regeneration and tumorigenesis remain incompletely understood, it is clear that chromatin reorganization and epigenetic alterations contribute to plasticity, self renewal, and other stem cell properties (4, 11).

As a highly regenerative tissue and frequent site for cancer, the colon epithelium provides a unique paradigm to study plasticity and cell state during tumorigenesis. Colon epithelium comprises an intricately organized hierarchy of epithelial cells maintained by proliferative stem cells that reside at the base of crypts (3, 4, 6–8). Moreover, it is among the most regenerative tissues of the body, renewing itself every 3–5 days to maintain nutrient absorption essential for life and provide a protective barrier from gut pathogens and toxins. Stem cells at the crypt base in colon and small intestinal epithelium are marked by the serpentine coreceptor for Wnt signals, leucine-rich, repeat-containing G-protein–coupled receptor 5 (LGR5) (7, 8). Recent studies in murine small intestine also identified distinct populations of upper crypt cells, marked by fibroblast growth factor binding protein 1 (FGFBP1) or LGR4, that regenerate all lineages, including LGR5+ cells, the latter of which requires the Wnt agonist, R-spondin (12–14). In both small intestine and colon, a Wnt gradient maintains LGR5+ cells by repressing differentiation at the base where Wnt levels are highest, while allowing cells to differentiate as they move up the crypt with decreasing Wnt. Thus, tightly regulated Wnt signaling is fundamental to epithelial regeneration in the gut.

Not surprisingly, mutations that activate Wnt signals are common in colon adenomas and adenocarcinomas (15–22). Inactivating mutations in the gene encoding the Adenomatosis Polyposis Coli (APC) tumor suppressor protein, first described in the familial adenomatosis polyposis (FAP) syndrome, are the most common genetic lesions found in colon adenomas and carcinomas (15–22). APC normally restrains Wnt function by maintaining β-catenin in the cytoplasm, thereby preventing β-catenin entry into the nucleus to activate Wnt target genes together with the TCF-4 transcription factor. Genomic studies established a model whereby colon carcinomas develop from polyps harboring APC mutations after the stepwise accumulation of mutations that inactivate additional tumor suppressor genes and/or activate protooncogenes (15–16). Neoplastic polyps are thought to arise in colon stem cells expressing *LGR5*, although targeting mutated LGR5+ cells in therapy has not been feasible (7, 8). Importantly, colon cancer is the third leading cause of cancer-related deaths in the US, and the incidence is rising globally, particularly in younger individuals (23–26). Thus, studies are warranted to decipher changes in cell state occurring during colon tumorigenesis to identify mechanisms that could be targeted to intercept the transition from mutant cells and localized tumors to advanced disease.

High Mobility Group A (HMGA1) proteins are architectural transcription factors that bind AT-rich sequences where they modulate chromatin structure and gene expression (4, 27–55). The *HMGA1* gene is highly expressed during embryogenesis (4, 9, 34, 37) and in adult stem cells (4, 29, 30, 47), but is silenced in most differentiated cells. *HMGA1* becomes reexpressed in aggressive cancer cells and high levels portend adverse clinical outcomes (28–38, 40–43, 56–64). In colon cancer, *HMGA1* is among the genes most highly overexpressed compared with nonmalignant colon epithelium (4, 30, 57, 62). While mechanisms upregulating *HMGA1* in cancer are incompletely understood, increasing evidence suggests that diverse oncogenic pathways, including growth factors (65, 66), mutations — such as mutant *Apc* (67, 68), *KRAS* (28, 36, 69), or mutant *JAK2* (29) — and oncogenic transcription factors, like cMYC (70) or cJUN (71, 72), converge on *HMGA1* to induce its expression in distinct settings. In transgenic mouse models, *Hmga1* overexpression leads to tumorigenesis (35, 41, 42, 58, 64). For example, transgenic mice overexpressing *Hmga1* in lymphoid cells develop clonal expansion with evolution to leukemia by upregulating transcriptional networks active in proliferating stem cells, poorly differentiated cancer cells, and inflammation (37, 38, 41, 58, 64). In experimental models of pancreatic cancer and myeloproliferative neoplasms, HMGA1 activates gene networks in a cell-intrinsic fashion to drive aberrant proliferation and differentiation, while inducing signals within the tumor microenvironment that promote fibrosis, culminating in tumor progression (28, 29). HMGA1 also upregulates genes involved in an epithelial-to-mesenchymal transition in colon cancer cell lines (35). In small intestinal stem cells, HMGA1 induces *Sox9* and Wnt signals from the stromal and epithelial niches to maintain the stem cell compartment and Paneth cell niche during epithelial regeneration (4, 30). However, HMGA1 function in colon stem and progenitor cells during tumorigenesis was previously unknown.

Here, we uncover a previously unknown role for HMGA1 in modulating transcriptional networks to expand LGR5+ stem cells and Paneth-like cells during tumorigenesis driven by *Apc* deficiency. Strikingly, loss of just a single *Hmga1* allele disrupts tumorigenesis while prolonging survival in two different models of colon tumorigenesis with APC inactivation, including mice with biallelic deletion of colonic epithelial *Apc* (*CDX2P-CreER^T2^Apc^fl/fl^*) (73, 74) and mice harboring monoallelic mutant *Apc* (*Apc^Min/–^* or Min mice) colonized with the inflammatory human symbiote, enterotoxigenic *Bacteroides fragilis* (ETBF) (75–79). Single cell RNA sequencing (scRNA-seq) in *Apc*-deficient crypt epithelium reveals that HMGA1 maintains colon crypt cells in a stem and Paneth-like cell state while depleting differentiated enterocytes. Integration of transcriptomic analyses with assays of chromatin accessibility demonstrate that HMGA1 activates Wnt signals by “opening” chromatin at gene loci governing Wnt signaling, including the stem cell regulator, *Achaete-Scute Family BHLH Transcription Factor 2* (*Ascl2*)*,* in addition to Wnt agonist receptors (*Lgr5, Lrp5*) and downstream effectors. We focus on the gene encoding ASCL2 as a master regulator of cell fate in the small intestine, although its role in the colon was previously unknown. In human colon cancer cells, HMGA1 directly induces *ASCL2* by recruiting activating histone marks. Further, silencing *HMGA1* disrupts oncogenic properties (proliferation and clonogenicity), while reexpression of *ASCL2* partially rescues oncogenic phenotypes in HMGA1-depleted human colon cancer cells. Most importantly, both *HMGA1* and *ASCL2* are coexpressed and upregulated in human colon cancer. Surprisingly, HMGA1 depletion has minimal effects on colon epithelial regeneration under homeostatic conditions. Our results establish HMGA1 as an epigenetic gatekeeper of *ASCL2* and Wnt signals in colon stem cells during tumorigenesis, but not steady state homeostasis, highlighting HMGA1 pathways as promising therapeutic targets for colon carcinogenesis.

## Results

### Loss of just a single Hmga1 allele is sufficient to decrease tumorigenesis and prolong survival in mice with colon tumors driven by biallelic Apc inactivation.

**Figure 1:**
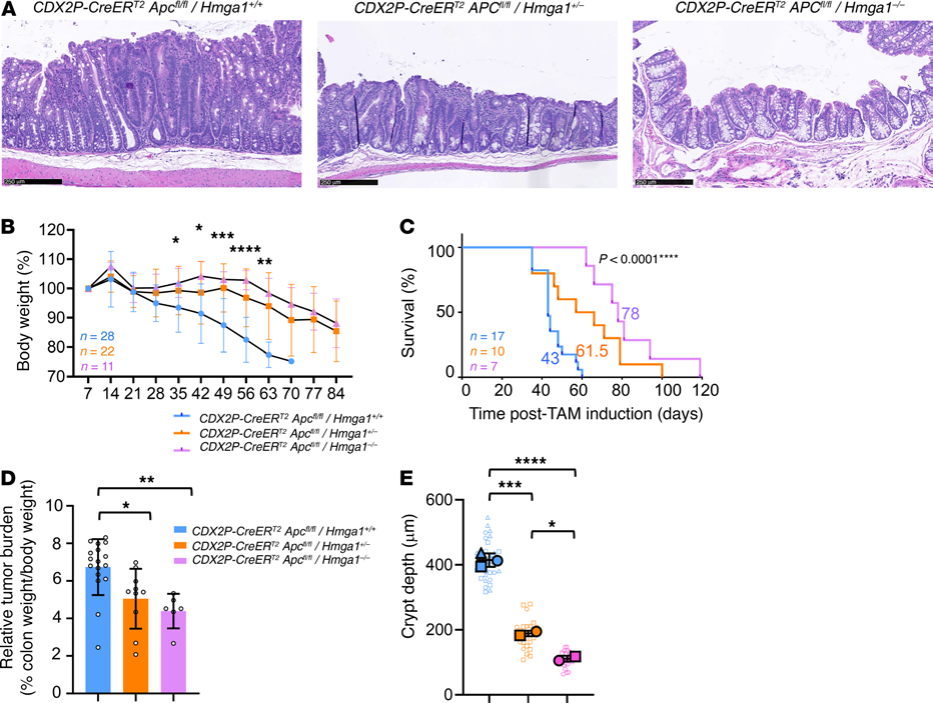
Loss of a single *Hmga1* allele mitigates colon tumorigenesis and prolongs survival in *CDX2P-CreER^T2^/Apc^fl/fl^* mice. (**A**) Representative images (H&E) of proximal colon in *CDX2P-CreER^T2^/Apc^fl/fl^* mice with *Hmga1* intact (*Hmga1^+/+^* top), heterozygous deletion (*Hmga1^+/–^*, middle), or homozygous deletion (*Hmga1^–/–^*, bottom) at survival endpoint necropsy (top: day 35 after TAM; middle: day 57; bottom: day 81). Scale bars: 250 μm. (**B**) Relative weight changes in *CDX2P-CreER^T2^/Apc^fl/fl^* models after TAM. (*P < 0.05, **P < 0.01, ***P < 0.001, ****P < 0.0001, 1-way ANOVA). (**C**) Kaplan-Meier plot showing survival in *CDX2P-CreER^T2^/Apc^fl/fl^* mice with *Hmga1^+/+^*, *Hmga1^+/–^*, or *Hmga1^+/+^*. (****P < 0.0001; Mantel-Cox test). (**D**) Relative colon weight to body weight in *CDX2P-CreER^T2^/Apc^fl/fl^* mice with *Hmga1^+/+^*, *Hmga1^+/–^*, or *Hmga1^–/–^* (*P < 0.05; *Hmga1^+/+^* versus *Hmga1^+/–^*, **P < 0.01, *Hmga1^+/+^* versus *Hmga1^–/–^*; Tukey’s multiple comparison test following significance by 1-way ANOVA). (**E**) Proximal colon crypt depth in *CDX2P-CreER^T2^/Apc^fl/fl^* models (*P < 0.05, ***P < 0.01, ****P < 0.0001; 1-way ANOVA with Tukey’s multiple comparison test). Each shape (circle, square, triangle) corresponds to a different mouse (*n* = 2–3/genotype). The solid shapes show the mean from each mouse; the open, smaller shapes represent individual measurements/crypt (range = 9–13 crypts/mouse) at × 20 magnification.

Because previous studies from our group and others showed that *HMGA1* is highly overexpressed in human colon cancer (30, 35, 62) and required for oncogenic properties in colon cancer cell lines (35), we sought to assess its role in colon tumorigenesis in vivo. Since *APC* is the most commonly mutated gene in human colon cancer (15), we examined *CDX2P-CreER^T2^Apc^fl/fl^* mice, an established model of colon tumorigenesis caused by inducible, biallelic loss of *Apc* within colon epithelium (73, 74). *CDX2P-CreER^T2^Apc^fl/fl^* mice were crossed to mice with global *Hmga1* deficiency (heterozygous or homozygous). Importantly, mice with heterozygous *Hmga1* have normal development and lifespans, whereas those with homozygous deficiency have partial embryonic lethality and develop premature aging phenotypes (kyphosis, bone loss, greying, and shortened lifespans) beginning after 10–12 months of age (29, 56). As expected, *CDX2P-CreER^T2^Apc^fl/fl^* mice with *Hmga1* heterozygous or homozygous deficiency have lower *Hmga1* gene expression and protein levels in colon epithelium (Supplemental Figure 1, A and B; supplemental material available online with this article; https://doi.org/10.1172/JCI184442DS1). Following induction of Cre recombinase-mediated *Apc* deletion by tamoxifen (TAM), *CDX2P-CreER^T2^Apc^fl/fl^* mice with intact *Hmga1* alleles develop epithelial hyperplasia in the cecum, proximal, and midcolon regions (Figure 1A) associated with weight loss by 4 weeks (Figure 1B) and decreased survival (median survival 43 days after TAM; *n* = 16) (Figure 1C). Strikingly, loss of just a single *Hmga1* allele in *CDX2P-CreER^T2^Apc^fl/fl^* mice mitigates weight loss while prolonging survival (median survival 61.5 days; *P* < 0.0001, *n* = 12; Figure 1, A–C). Survival is prolonged further (median survival 78 days; *P* < 0.0001, n = 7) (Figure 1C) in mice with *Hmga1* homozygous deficiency, indicating that *Hmga1* gene dosage modulates tumor progression in this model.

**Figure 2:**
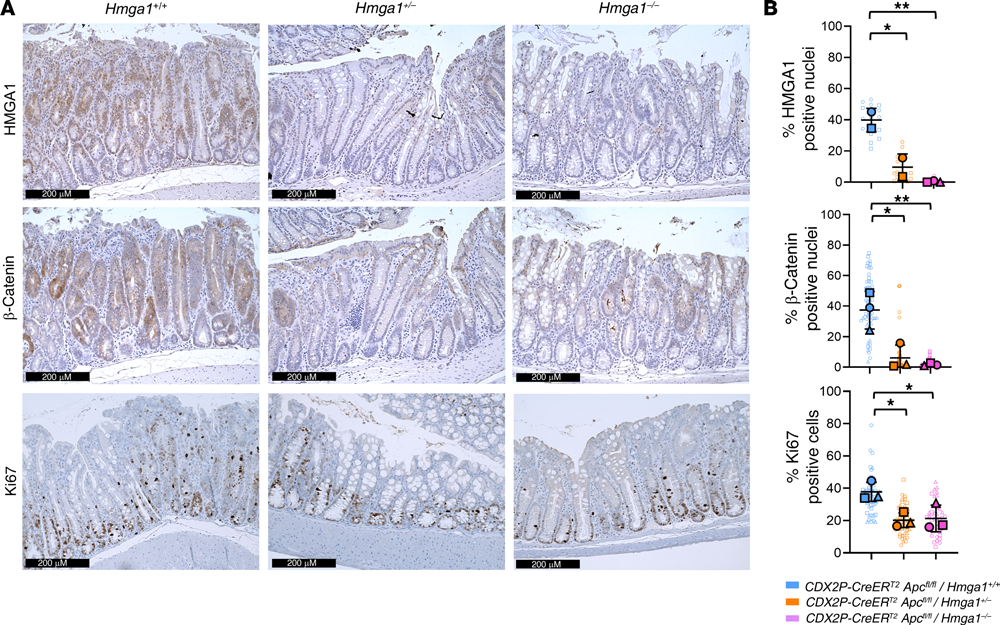
*Hmga1* haploinsufficiency decreases β-catenin and Ki67 in *CDX2P-CreER^T2^/Apc^fl/fl^* mice. (**A**) Representative IHC images of nuclear HMGA1 (top), β-catenin (middle), and Ki67 (bottom) in *CDX2P-CreER^T2^/Apc^fl/fl^* models at 3 weeks after TAM. Scale bar: 200 μm. (**B**) Quantitative comparisons of IHC images (*P < 0.05, **P < 0.01; Tukey’s multiple comparison test following significance by 1-way ANOVA). Each shape (circle, square, triangle) corresponds to a different mouse [top bar graph (*n* = 2–3/genotype), middle bar graph (*n* = 3/genotype), bottom bar graph (*n* = 3/genotype)]. The solid shapes show the mean from each mouse; the open, smaller shapes represent individual values/field (range = 8–24 fields/mouse) at × 20 magnification.

**Figure 3:**
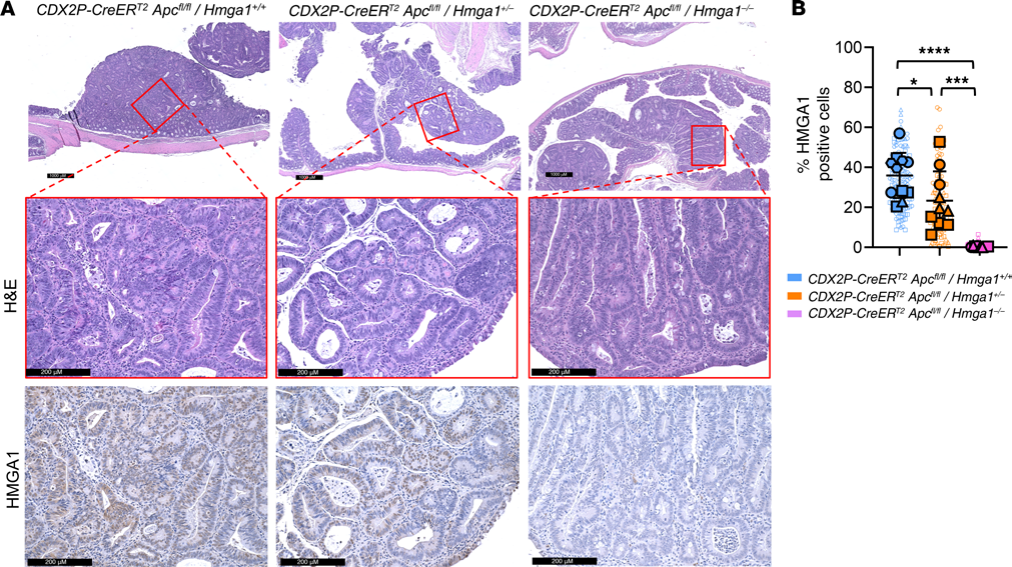
*Hmga1* deficiency decreases colon tumorigenesis. (**A**) Representative IHC images of nuclear HMGA1 (top), β-catenin (middle), and Ki67 (bottom) in *CDX2P-CreER^T2^/Apc^fl/fl^* models at 3 weeks after TAM. Scale bars: 1,000 μm (top panel); 200 μm (lower panel). (**B**) Quantitative comparisons of IHC images (*P < 0.05, ***P < 0.001, ****P < 0.0001; Tukey’s multiple comparison test following significance by 1-way ANOVA). Each shape (circle, square, triangle, hexagon) corresponds to a different mouse (*n* = 3–4/genotype). The solid shapes show the mean values from each tumor; the open, smaller shapes represent individual values/field (range = 6–17 fields/tumor from 1–5 tumors/mouse) at × 20 magnification.

To determine more precisely how HMGA1 modulates tumorigenesis in *CDX2P-CreER^T2^Apc^fl/fl^* mice, we compared colon weights as a surrogate for tumor burden since extensive, contiguous tumors in the proximal colon precludes precise enumeration. Both the absolute and relative colon weight (% colon weight/body weight) increase in *CDX2P-CreER^T2^Apc^fl/fl^* mice with intact *Hmga1* compared with those with heterozygous or homozygous *Hmga1* deficiency (Figure 1D and Supplemental Figure 1C). At 21 days following *Apc* inactivation, colon epithelium becomes thickened and dysplastic with extensive adenomatous changes and increased crypt depth in *CDX2P-CreER^T2^Apc^fl/fl^* mice with intact *Hmga1* (Figure 1, A and E, and Figure 2, A and B). Intranuclear HMGA1 is prominent throughout the crypts up to the luminal enterocytes in *CDX2P-CreER^T2^Apc^fl/fl^* mice with intact *Hmga1* by IHC. By contrast, intranuclear HMGA1 is normally restricted to the crypt bases in WT mice lacking *Apc*-deficiency (Supplemental Figure 1D). Since APC restrains Wnt signals by sequestering β-catenin within inhibitory, cytoplasmic complexes, we compared β-catenin levels and localization in nuclei and cytoplasm in the Apc-deficient models. Following Apc inactivation (day 21), both nuclear and cytoplasmic β-catenin levels increase in colon epithelial cells of mice with intact HMGA1 compared with those with heterozygous or homozygous Hmga1 deficiency, paralleling the distribution of intranuclear HMGA1 (Figure 2, A and B, and Supplemental Figure 1E). Cells staining positive for the proliferation marker Ki67 are also increased in *CDX2P-CreER^T2^Apc^fl/fl^* mice with intact HMGA1 compared with those with HMGA1 deficiency early in tumorigenesis, although they predominate at the crypt bases (Figure 2, A and B). In addition, HMGA1 increases in the adenomatous epithelium compared with nontumor, midcolon epithelium in mice with intact HMGA1 (Figure 3, A and B, and Supplemental Figure 1, A and B). Intriguingly, HMGA1 also increases in tumors from mice with *Apc* inactivation and *Hmga1* heterozygous deficiency compared with adjacent nontumor colon epithelium (Figure 3, A and B, and Supplemental Figure 1, A and B). These findings indicate that loss of just a single *Hmga1* allele in the setting to *Apc* inactivation decreases hyperproliferation, β-catenin levels, and tumorigenesis.

### HMGA1 hemizygous deficiency mitigates colon tumorigenesis induced by ETBF in Min mice.

**Figure 4:**
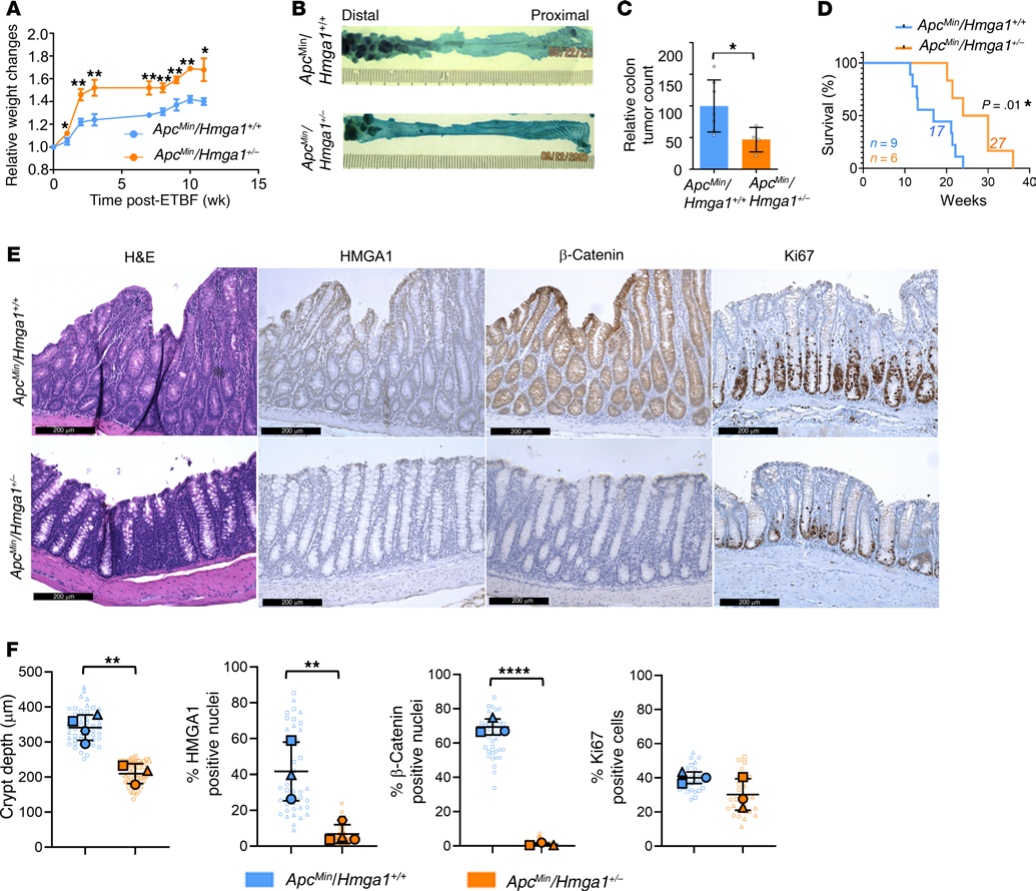
*Hmga1* haploinsufficiency disrupts colon tumorigenesis induced by ETBF in *APC^Min/+^* mice. (**A**) Body weights at necropsy after ETBF in *Apc^Min/+^* mice with intact *Hmga1* or heterozygous *Hmga1* (*P < 0.05, **P < 0.01, ***P < 0.001; student’s *t* test). (**B**) Representative images of methylene-blue stained colons to visualize tumors in *Apc^Min/+^/Hmga1^+/+^* mouse (top) compared with *Apc^Min/+^/Hmga1^+/–^* mouse (bottom) at 11–12 weeks after ETBF. (**C**) Normalized tumor numbers in *Apc^Min/+^* models (*P < 0.05; Mann-Whitney test). (**D**) Kaplan-Meier plot showing survival in in *Apc^Min/+^* mice with intact *Hmga1* or heterozygous (*P < 0.05; Mantel-Cox test). (**E**) Representative images (H&E left; IHC right; Scale bars: 200 μm) for HMGA1 (second column), β-catenin (third column), and Ki67 (right) in distal colon of *Apc^Min/+^* models at 11–12 weeks after ETBF. (**F**) Comparison of crypt depths (**P < 0.01) and IHC for nuclear HMGA1 (**P < 0.01), nuclear β-catenin (****P < 0.0001) and Ki-67(*P* = 0.16, unpaired student’s *t* test for each comparison) in *Apc^Min/+^* models. For crypt depth (left), each shape (circle, square, triangle, hexagon) corresponds to a different mouse (*n* = 3–4/genotype). The solid shapes show the mean from each mouse; the open, smaller shapes represent individual measurements/crypt (range = 9–16 crypts/mouse). For the IHC comparisons, each shape (circle, square, triangle, hexagon) corresponds to a different mouse (*n* = 3–4/genotype), the solid shapes show the mean value from each mouse; the open, smaller shapes represent individual values/field (range=9-19 fields/mouse) at x20 magnification.

**Figure 5:**
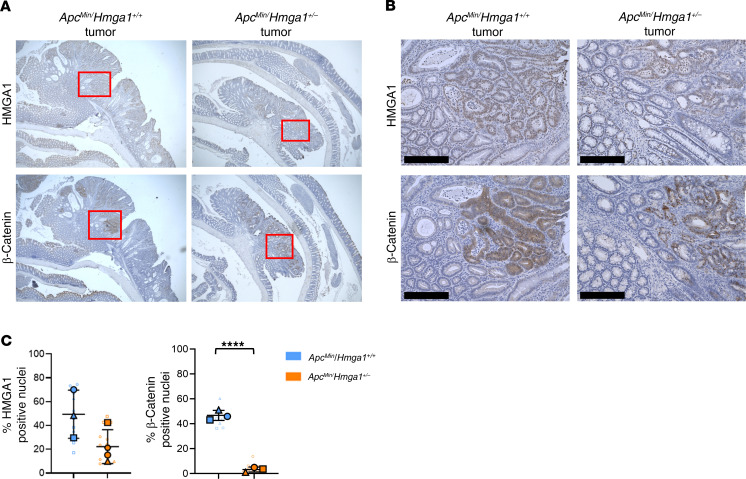
*Hmga1* haploinsufficiency decreases β-catenin and tumorigenesis induced by ETBF in *APC^Min/+^* mice. (**A**) Representative images (H&E) of distal colon tumors in *Apc^Min/+^* models at 11–12 weeks after ETBF. (**B**) Representative images (IHC) of distal tumors in *Apc^Min/+^* models for HMGA1 and β-catenin at 11–12 weeks after ETBF. Scale bars: 200 μm. (**C**) Quantitative IHC comparisons of distal tumors in *Apc^Min/+^* models for HMGA1 (*P =* 0.09) and β-catenin (****P < 0.0001; unpaired student’s *t* test for both). Each shape (circle, square, triangle, hexagon) corresponds to a different mouse (*n* = 3–4/genotype). The solid shapes show the mean from each mouse; the open, smaller shapes represent individual values/field (range = 2–4 tumor fields/mouse) at × 20 magnification.

Next, we investigated HMGA1 function in the multiple intestinal neoplasia (Min) model, which harbors a heterozygous *Apc* loss-of-function mutation (*Apc^Min/+^* or *Min^+/–^*) and develops distal colon tumors following inoculation with the human symbiotic bacterium ETBF. This model recapitulates salient features of human colon tumors with respect to the mutational status, location in the distal colon, and histopathology (75–79). Furthermore, ETBF colonization is common in colon cancer (up to 90%) and epidemiologic studies suggest that it increases the risk of carcinogenesis (75–85). Following inoculation with ETBF at 5–6 weeks of age, Min mice with intact *Hmga1* exhibit poor weight gain and robust distal colon tumorigenesis by 11–12 weeks with a median survival of 17 weeks; by contrast, Min mice with global *Hmga1* hemizygosity gain more weight, develop fewer tumors, and exhibit prolonged survival (Figure 4, A–D). Histologic examination shows hyperproliferative colon epithelium and adenomatosis with increased crypt depth in the distal colons of Min mice with intact HMGA1 compared with those with HMGA1 deficiency (Figure 4, E and F). As expected, HMGA1 mRNA and protein levels by IHC are increased in mice with intact HMGA1 compared with *Hmga1* haploinsufficient Min mice (Figure 4, E and F, Figure 5, A–C, and Supplemental Figure 2A). Similarly, the proportion of cells staining positive for intranuclear β-catenin and cytoplasmic β-catenin are greater in colon epithelium and tumors of *Apc^Min/+^* mice with intact HMGA1, although Ki67 was unchanged in mice with intact or haploinsufficient HMGA1 (Figure 4, E and F, and Supplemental Figure 2B). Intriguingly, HMGA1 protein staining is similar in tumors from *Apc^Min/+^* mice with intact HMGA1 and HMGA1 haploinsufficiency, suggesting that mice with HMGA1 haploinsufficiency can upregulate the intact *Hmga1* allele to increase HMGA1 levels within their tumors (Figure 5, A–C). The small intestinal tumor burden is also greater in *Apc^Min/+^* mice with intact HMGA1 (Supplemental Figure 2C), indicating that HMGA1 contributes to tumorigenesis in both the colon and small intestine of Min mice.

### Loss of a single Hmga1 allele within the colon epithelium is sufficient to reduce colon tumorigenesis induced by ETBF in Min mice.

**Figure 6:**
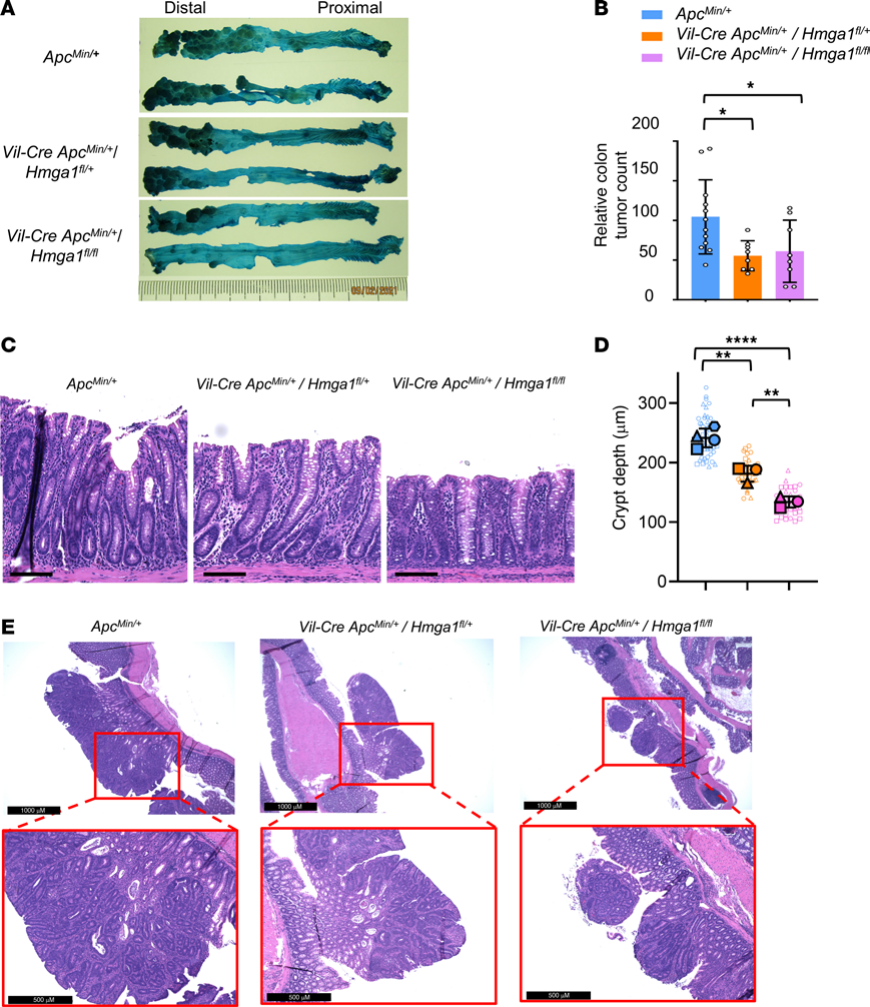
Loss of *Hmga1* allele within colon epithelium decreases colon tumorigenesis induced by ETBF in *Apc^Min/+^* mice. (**A**) Representative images of methylene-blue–stained colons of *Apc^Min/+^* mice (top) compared with *Apc^Min^* with tissue-specific heterozygous *Hmga1* deletion (middle) and tissue-specific homozygous *Hmga1* deletion (bottom) at 11–12 weeks after ETBF. (**B**) Relative tumor numbers (%) in *Apc^Min^* mice with intact *Hmga1,* tissue-specific heterozygous *Hmga1* deletion, or tissue-specific homozygous *Hmga1* deletion from 3 separate experiments; tumor numbers in control were assigned a value of 100 (*P < 0.05; Mann-Whitney test for both comparisons). (**C**) Representative images (H&E) of distal colon of *Apc^Min/+^* with or without tissue-specific *Hmga1* deficiency models. Scale bars: 100 μm. (**D**) Distal colon crypt depths in *Apc^Min/+^* mice with or without tissue-specific *Hmga1* deficiency. (**P < 0.01, ****P < 0.0001; Tukey’s multiple comparisons test following significance by 1-way ANOVA). Each shape (circle, square, triangle, hexagon) corresponds to a different mouse (n = 3–4/genotype). The solid shapes show the mean value from each mouse; the open, smaller shapes represent individual measurements/crypt (range = 9–19 crypts/mouse) at × 20 magnification. (**E**) Representative images (H&E) of distal colon tumors of *Apc^Min/+^* with or without tissue-specific *Hmga1* deficiency.

To determine whether HMGA1 deficiency within colon epithelium is sufficient to mitigate tumorigenesis in the Min-ETBF model, we generated Min mice with *Hmga1* deficiency (hetero- and homozygous genetic deletion) restricted to colon and small intestinal epithelium by crossing *Apc^Min/+^* mice with *Hmga1^fl/fl^* mice on a *Vil*-cre background. Notably, Min mice with tissue-specific HMGA1 deficiency developed fewer colon tumors and decreased crypt depth compared with Min mice with intact HMGA1 (Figure 6, A–E). Surprisingly, Min mice with *Hmga1* haploinsufficiency had a similar decrease in colon tumor number as Min mice with homozygous *Hmga1* loss, suggesting that a relatively modest decrease in HMGA1 within the epithelial compartment alone is sufficient to mitigate tumorigenesis. Small intestinal tumors also decrease modestly in this model, but only with homozygous loss of *Hmga1* (Supplemental Figure 3A). By contrast, tissue-specific biallelic loss of *Hmga1* in colon crypts from WT mice lacking *Apc* mutation show no significant changes in crypt depth, suggesting that HMGA1 deficiency under steady state, homeostatic conditions (no ETBF colonization) is not deleterious to colon epithelial regeneration (Supplemental Figure 3B). Together, these findings demonstrate that HMGA1 within the crypt epithelium drives tumorigenesis, and, moreover, tissue-specific, *Hmga1* haploinsufficiency is sufficient to impair colon tumor formation driven by mutant *Apc* and inflammatory ETBF, highlighting HMGA1 as a promising potential therapeutic target.

### HMGA1 expands colon stem cells and Paneth-like cells while depleting more differentiated enterocytes in Apc-deficient colon crypts.

**Figure 7:**
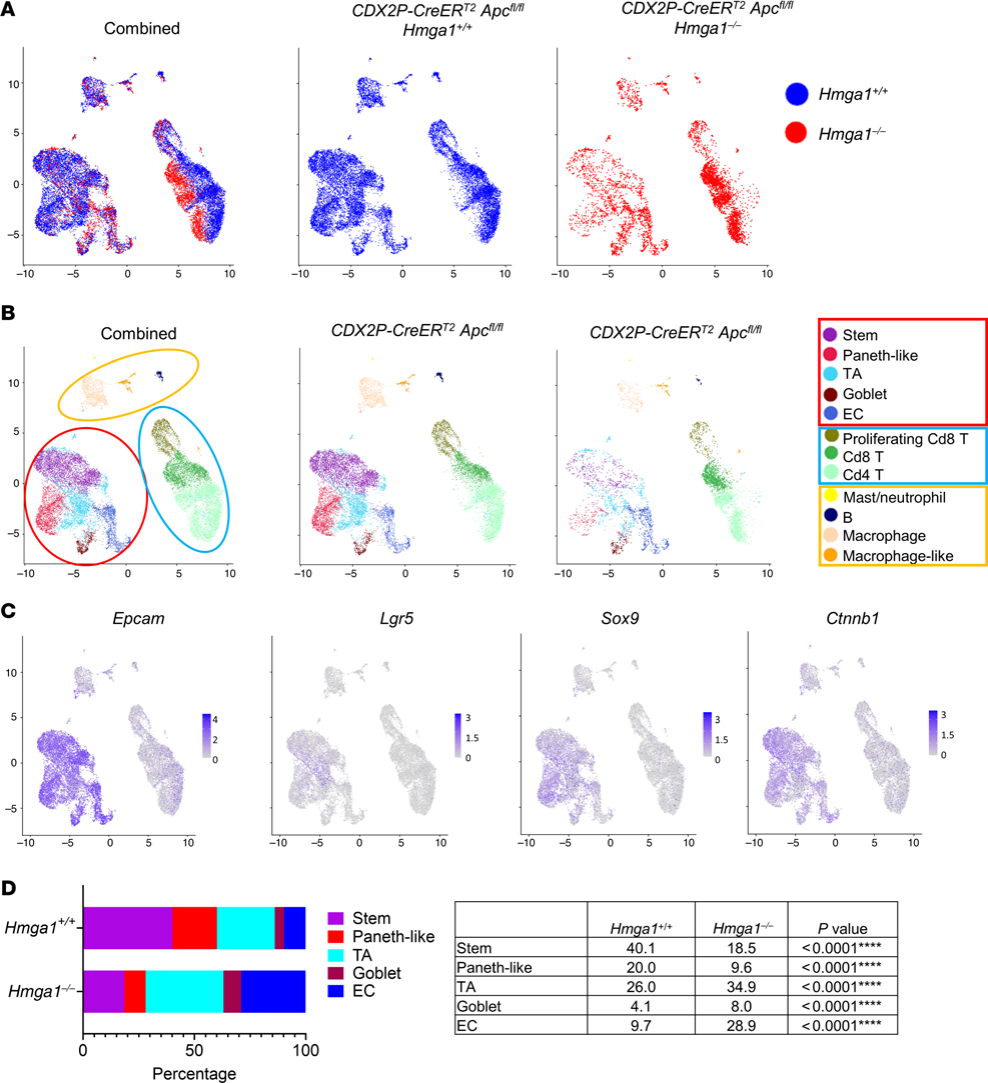
HMGA1 expands colon stem cells and Paneth-like cells while depleting more differentiated cells in *Apc-*deficient colon crypts. (**A**) UMAPs from scRNA-seq of crypt cells from *CDX2P-CreER^T2^ Apc^fl/fl^* mice with *Hmga1^+/+^* or *Hmga1^–/–^*; shown together (left) or separately to highlight differences (center and right). (**B**) UMAP from scRNA-seq by cluster. Three distinct islands capture epithelial cell types (red circle), T cells (blue circle), and other immune cells (yellow). Imputed cell identities are designated by separate colors. TA, transit amplifying cells; EC, enterocytes. (**C**) *Epcam*, *Lgr5,* and other Wnt genes (*Sox9, Ctnnb1*) are enriched in the epithelial island. Single cell transcripts from both genotypes are shown. (**D**) Relative proportion of cell types in crypt cells by genotype (bar graph, left; Table, right). (Association between cell and HMGA1 status was evaluated by χ^2^ test for each cell type versus all others).

To investigate molecular mechanisms underlying HMGA1 in *Apc-*deficient colon crypts, we performed scRNA-seq in proximal colon crypt cells from *CDX2P-CreER^T2^Apc^fl/fl^* mice with intact *Hmga1* compared with those with heterozygous or homozygous *Hmga1* deficiency. We examined transcriptomes at early stages in tumorigenesis (21 days following *Apc* inactivation via TAM) to identify mechanisms involved in tumor initiation. Single-cell transcriptomes depicted by uniform manifold approximation and projection (UMAP) reveal differences in overall distribution in cells with or without HMGA1 (Figure 7A). Unsupervised hierarchical clustering of transcripts (via Seurat) revealed 12 clusters (Figure 7B) from which cell identities were imputed using established markers (Supplemental Table 1). Of these clusters, five are comprised of epithelial crypt cells (denoted epithelial island) based on expression of the colon epithelial cell adhesion marker gene (*Epcam*), colon stem and progenitor cell genes (*Lgr5*, *Sox9*, *Ctnnb1*), and proximity by UMAP (Figure 7C). The remaining clusters are comprised of immune cells (Figure 7B). Within the immune cell islands, we identified Cd4^+^ and Cd8^+^ T cells with smaller populations of B cells, macrophages, and other myeloid lineages (macrophage-like, mast cells, and neutrophils) (Supplemental Figure 4A).

To dissect HMGA1-dependent changes in the cell of origin for colon tumors, we focused on the epithelial island. With intact HMGA1, the LGR5^+^ stem cell population, defined by high levels of *Lgr5, Msi1, Bmi1,* and other stem cell transcripts (Supplemental Table 1)*,* comprise the majority of cells (40.1%) within *CDX2P-CreER^T2^Apc^fl/fl^* colon crypts (Figure 7D). Transit amplifying (TA) cells are the next most abundant population, constituting 26% of epithelial crypt cells, whereas Paneth-like cells, based on Paneth cell markers (*Lyz1, Mmp7, Sox9, Retnlb, Chil3, Reg3g,* and *Deta;* Supplemental Table 1), comprise 20% of crypt cells. Intriguingly, while Paneth cells are not present in normal colon epithelium, Paneth cell “metaplasia” has been reported in proximal colon epithelium in adenomas, adenocarcinoma, and inflammatory bowel disease, and ectopic Paneth cells were observed in colon epithelium of *Apc*-deficient mouse models (73, 74, 85, 86). The terminally differentiated enterocyte (EC) and goblet cell clusters comprise the least frequent crypt cell types in this model (9.7% and 4.1%, respectively). Strikingly, HMGA1 deficiency decreases the proportion of stem and Paneth-like cell clusters by about 50% (*P <* 0.0001) together with a concurrent expansion in the proportion of differentiated ECs (from 9.7% to 28.9%; *P <* 0.0001), TA cells (from 26% to 34.9%; *P <* 0.0001), and goblet cells (from 4.1% to 8%; *P <* 0.0001) within the crypt epithelium. In *Apc-*deficient crypts with *Hmga1* heterozygous deficiency, the changes in most clusters are intermediate between crypts with intact or homozygous deficiency of *Hmga1* (Supplemental Figure 4, B and C). Together, these results indicate that intact HMGA1 is required to maintain the colon stem and Paneth-like cells in the setting of *Apc* deficiency while depleting more differentiated cells (ECs and goblet cells).

Within the immune cell islands, Cd4^+^ and Cd8^+^ T cells increase in frequency in the HMGA1-deficient crypt cells (Supplemental Figure 4), the latter of which could reflect an increase in tumor-infiltrating T lymphocytes. Both Cd4^+^ and Cd8^+^ T cells with *Hmga1* genetic deletion also exhibit a shift on UMAP, indicating that HMGA1 loss within these T cell populations alters their underlying transcriptomes (Supplemental Figure 4A).

### Trajectory and cell state analyses show that HMGA1 maintains an earlier cell state in Apc-deficient crypt cells.

**Figure 8:**
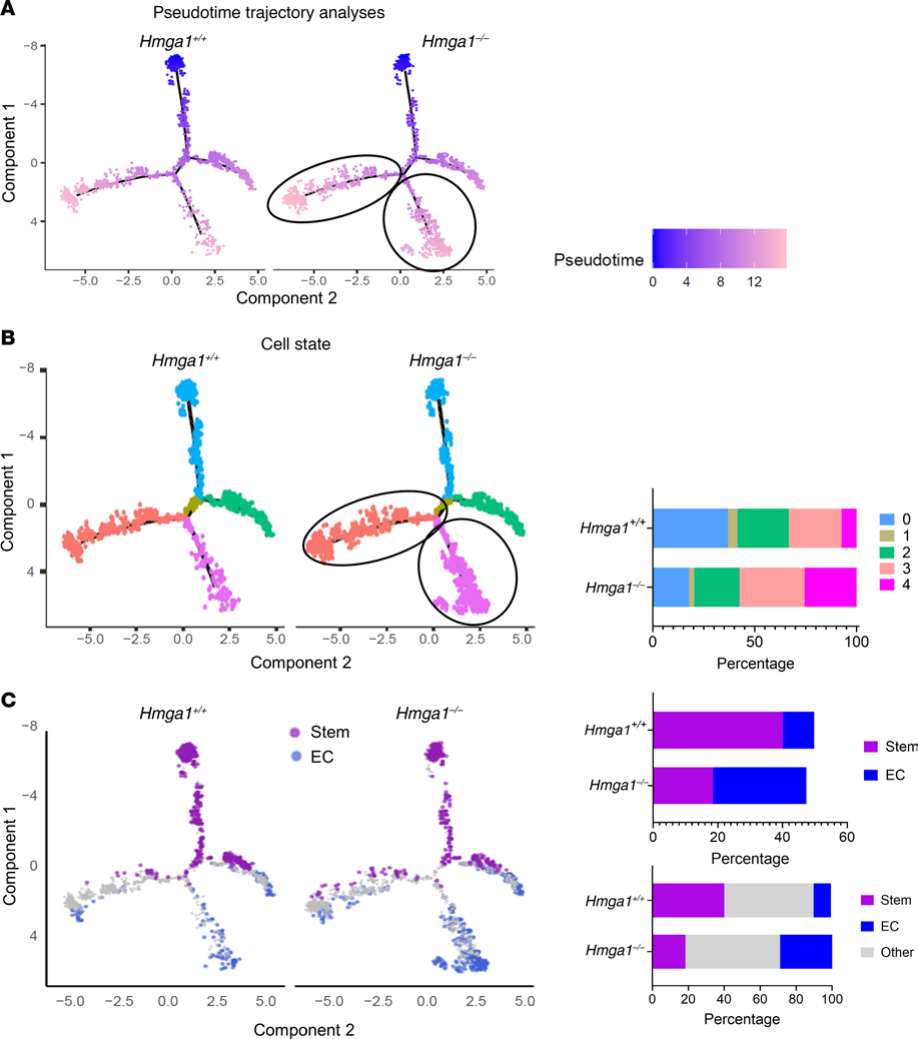
*Hmga1* deficiency alters cell state, decreasing stem and Paneth-like cell populations while expanding more differentiated cell populations in *Apc* deficient crypt cells. (**A**) Pseudotime trajectory analysis estimated from scRNA-seq of *CDX2P-CreER^T2^ Apc^fl/fl^* crypt cells from the epithelial island with *Hmga1*^+/+^ or *Hmga1^–/–^*. HMGA1 deficient cells are more prominent in later stages of pseudotime (indicated by black ovals) compared with time 0 cells. (**B**) Cell states defined by the top 200 most differentially expressed genes on the trajectories from pseudotime analysis were assigned 0–4 and indicated by color on a trajectory plot (left) or bar graph (right). Note the skewing to cell states 3 and 4 in HMGA1 deficient cells. (**C**) Stem cells and enterocytes (ECs) imputed from scRNA-seq are shown on the trajectories to highlight the major differences between *CDX2P-CreER^T2^ Apc^fl/fl^* cells with intact HMGA1 or HMGA1 deficiency. HMGA1 deficient cells have increased ECs (blue) with decreased stem cells (violet). Bar graphs show relative cell frequencies (right); the top graphs show only stem and ECs, the bottom includes all cells with grey depicting cells that are not stem cells nor ECs.

To delineate HMGA1 function in differentiation dynamics in *Apc-*deficient colon crypt epithelium, we performed pseudotime trajectory analyses, assigning time = 0 to the most dedifferentiated, stem cell cluster (via Seurat, Monocle 2). Intact HMGA1 results in a greater proportion of cells at earlier stages in development (time = 0; undifferentiated stage) whereas HMGA1 deficiency leads to more cells in later stages (time = 12; more differentiated stage) within the differentiation trajectory (Figure 8A). Next, we applied cell state analysis (Seurat, Monocle 2) as a more static assessment of differentiation status of each cell along the trajectory in *Apc-*deficient epithelial crypt cells (Figure 8B). Cell states (defined by the top 200 most differentially expressed genes within 5 groups with distinct transcriptomes) were assigned to individual cells along the trajectory. Similar to our cluster analysis, *Apc-*deficient crypt cells with intact *Hmga1* include a greater proportion of cells in an undifferentiated stem cell state (state 0) or Paneth-like state (state 1) compared with those with HMGA1 deficiency, which skews development to later, more differentiated cell states (states 3–4) (Figure 8B) or ECs (Figure 8C).

### Single cell transcriptomes suggest that HMGA1 accelerates proliferation by inducing gene networks involved in cell cycle progression.

Next, we inferred cell cycle status of each cell in the epithelial cluster from scRNA-seq (Seurat; standard settings). Transcriptomic changes suggest that intact HMGA1 in *Apc-*deficient crypt epithelial cells function by increasing proliferation, as evidenced by decreases in the proportion of cells in G0/G1 concurrent with increases in the proportion reaching G2/M; the proportion of S phase cells were similar in *Apc-*deficient crypt cells with or without HMGA1 deficiency (Supplemental Figure 5). The proliferation marker gene encoding Ki67 is among the most upregulated genes of all G2/M genes. Collectively, our single-cell transcriptomes, together with increases in crypt depth, Ki67 protein staining, and tumorigenesis in mice with colon epithelial *Apc* inactivation and intact HMGA1 (Figure 1) are consistent with a model whereby HMGA1 increases proliferation restricted to cells at the earliest developmental stages, leading to expansion in stem and Paneth-like cells at the expense of more differentiated cells within the crypt epithelium.

### HMGA1 activates gene networks within crypt epithelial cells involved in IFN signaling, inflammation, DNA repair, proliferation, and Wnt signaling.

To elucidate mechanisms underlying HMGA1 in *Apc*-deficient crypt cells, we performed gene set enrichment analysis (GSEA; MSigDB) with Hallmark and Curated gene sets (87, 88). GSEA with transcripts from all clusters (epithelial and immune cells) reveal that HMGA1 upregulates heterogenous pathways, including those associated with metabolism (oxidative phosphorylation and glycolysis), proliferation (MYC Targets V1 and MYC Targets V2), and inflammation (IFN-α Response and IFN-γ Response) (Supplemental Figure 6). HMGA1 also activates multiple WNT networks, including Wnt Pathway requiring MYC, Degradation of the β-catenin Destruction Complex, TCF Dependent Signaling in Response to WNT, and APC Targets (Table 1). By contrast, transcriptional networks repressed by HMGA1 include allograft rejection, IL2-STAT5 signaling, and mitotic spindle genes (Hallmark). Notably, repression in allograft rejection and IL2-STAT5 gene networks have been implicated in immune escape and decreases in cytotoxic tumor infiltrating lymphocytes (Supplemental Figure 6) (89). The heterogeneity in these pathways is consistent with the diverse cell populations (epithelial and immune) within the crypts.

**Figure 9:**
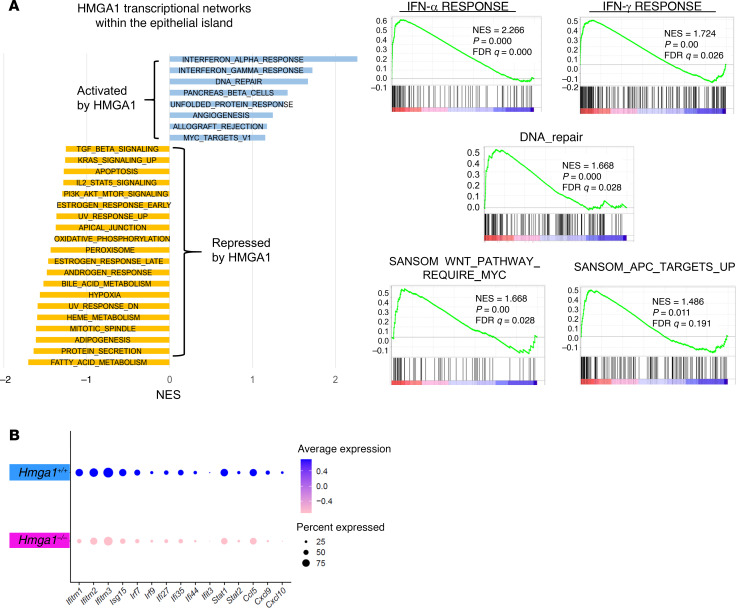
HMGA1 activates gene networks within the crypt epithelial island involved in IFN signaling, inflammation, DNA repair, proliferation, and Wnt signaling. (**A**) GSEA analysis (left) of single cell transcripts from the epithelial island reveals that HMGA1 activates pathways involved in inflammation (IFN-α, IFN-γ), DNA repair, and proliferation (MYC) while repressing pathways active in differentiated ECs (fatty acid metabolism, protein secretion); FDR ≤ 0.25. Enrichment plots (right) show HMGA1 networks in more detail, including genes involved in inflammation (IFN-α), DNA repair, and Wnt signaling. Normalized enrichment score (NES) and normalized *P* values are indicated. (**B**) IFN-inducible genes that mediate inflammatory signals, including *IFN-induced transmembrane 1, 2, 3,* (*Ifitm1, 2, 3*) genes*, IFN stimulated gene 15* (*Isg15*)*, Stat1*, *Stat2*, and cytokines (*Ccl5, Cxcl9, Cxcl10*) are activated by HMGA1. Dot plots depict gene expression (–0.4 to +0.4) and the proportion of cells (25%–75%) expressing each transcript within the epithelial island.

To focus our analysis on the tumor-initiating cells, we performed GSEA exclusively on transcripts from the crypt epithelial island. Further, this island is comprised of the majority of cells from the crypt isolates and cell numbers are sufficient for pathway analyses. In the remaining immune islands, cell numbers were insufficient for further GSEA. Strikingly, HMGA1 activates transcriptional networks involved in inflammation, including IFN-α and IFN-γ response genes and proliferation (MYC targets V1) within the epithelial island (Figure 9A). DNA repair genes are also induced, which is a frequent transcriptional response when quiescent stem cells are triggered to cycle and proliferate (Figure 9A) (90–92). Among the IFN networks, multiple IFN-induced genes that mediate inflammatory signals are upregulated by HMGA1, including *IFN-induced transmembrane proteins 1, 2, 3* (*Ifitm 1–3*), *IFN stimulated gene 15* (*Isg15*), *Stat1*, *Stat2*, and cytokines (*Ccl5, Cxcl9/10*) (Figure 9B). Wnt pathway genes are also prominent among the networks activated by HMGA1 within the epithelial crypt cells (Figure 9A and Table 1). By contrast, HMGA1 represses gene networks involved in fatty acid metabolism and adipogenesis, metabolic pathways used extensively by differentiated ECs in intestinal epithelium (Figure 9A) (93). HMGA1 also represses genes controlling protein secretion (Figure 9A), an important cellular function of differentiated ECs, which secrete digestive enzymes (93). Together, our single-cell transcriptomes and pathway analysis demonstrate that HMGA1 expands the stem and Paneth-like cells while driving transcriptional networks involved in proliferation, inflammation, Wnt signaling, and DNA repair. Conversely, HMGA1 restrains differentiation and represses metabolic gene networks active in differentiated ECs.

### HMGA1 enhances chromatin accessibility at gene loci involved in proliferation, DNA repair, inflammation, and Wnt signaling.

**Figure 10:**
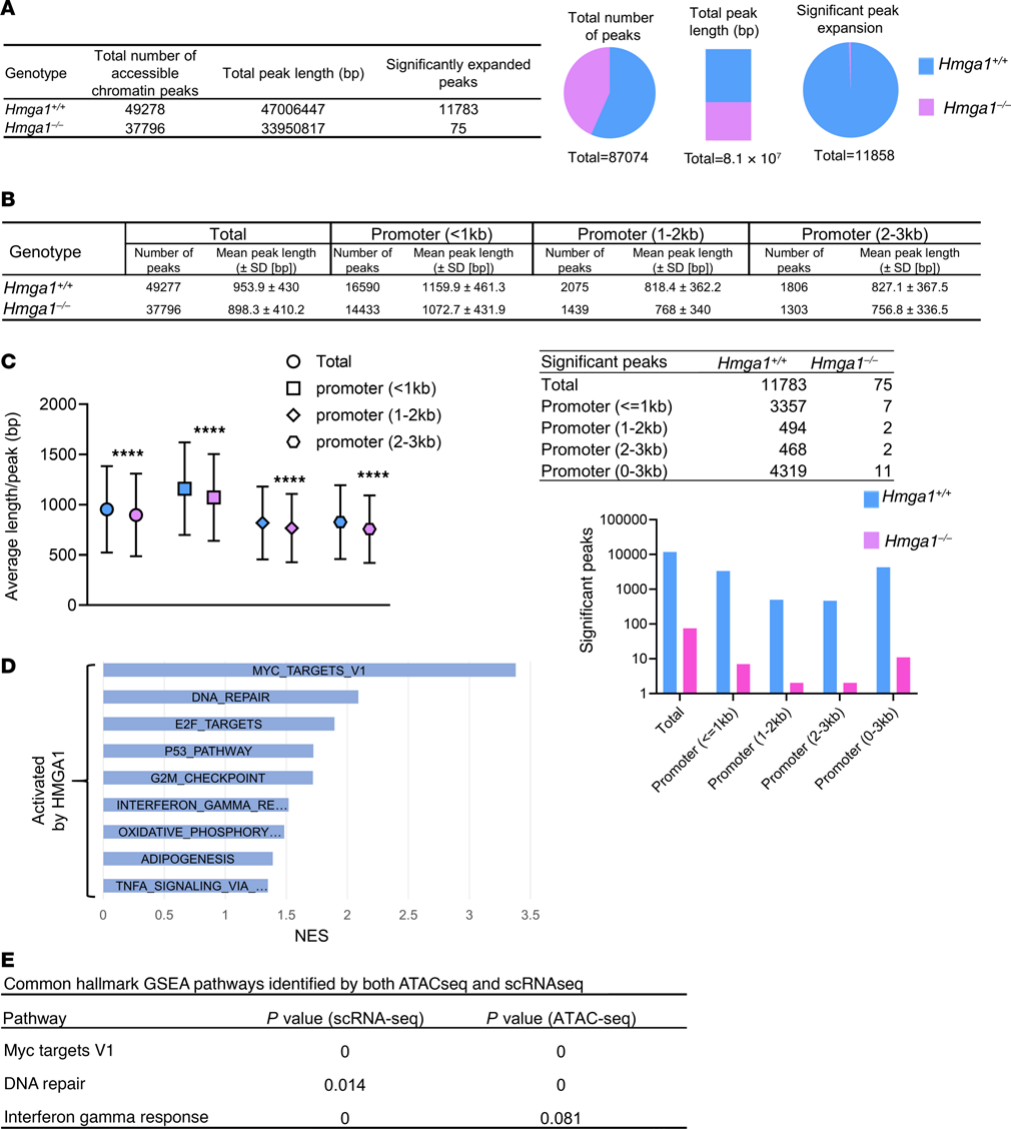
HMGA1 enhances chromatin accessibility at gene loci involved in proliferation, DNA repair, and inflammation. (**A**) HMGA1 increases chromatin accessibility in crypt cell nuclei globally in *CDX2P-CreER^T2^ Apc^fl/fl^* mice. (**B**) HMGA1 enhances chromatin accessibility in promoter regions ranging from 0 to –3 kb upstream of the transcription start sites shown by average peak lengths. (****P < 0.0001; student’s *t* test). (**C**) HMGA1 enhances chromatin accessibility in promoter regions ranging from 0 to –3 kb upstream of the transcription start sites shown by number of significantly expanded peaks. (*P* < 0.0001; χ^2^). (**D**) HMGA1 enhances chromatin accessibility in gene sets involved in proliferation, inflammation, and metabolism. (**E**) GSEA pathways identified by intersecting ATAC-seq and scRNA-seq pathways with associated *P* values.

Because HMGA1 is an architectural transcription factor that modulates chromatin structure, we performed assays to detect accessible chromatin mediated by HMGA1 via assays of transposase-accessible chromatin sequencing (ATAC-seq) in *Apc*-deficient crypt cells with or without HMGA1 deficiency (94). Notably, overall chromatin accessibility is enhanced in *Apc-*deficient crypt cells with intact HMGA1 compared with crypts lacking HMGA1 (Figure 10, A–C). HMGA1 results in both more peaks and longer stretches of accessible chromatin overall (Figure 10, A–C). Focusing on promoter regions (up to –3 kb from the transcription start sites), we also identified more peaks and longer stretches of open chromatin within these regulatory regions with intact HMGA1 (Figure 10, B and C). Similar to our scRNA-seq results, gene networks associated with HMGA1-mediated accessible chromatin included pathways involved in proliferation (MYC Targets V1, E2F Targets, G2M Checkpoint genes) and inflammation (IFN-γ, TNF-α signaling via NF-κB (Figure 10, D and E, and Supplemental Figure 7). Accessible chromatin is also enriched at Wnt signaling gene networks (Table 1). Intersecting pathways identified by both ATAC-seq and scRNA-seq (epithelial island) revealed that HMGA1 increased chromatin accessibility and expression of genes involved in proliferation (MYC Targets V1), DNA repair, inflammation (IFN-γ response genes), and Wnt signaling (Figure 10E and Table 1).

### HMGA1 amplifies expression of Wnt pathway genes in Apc-deficient colon crypts.

**Figure 11:**
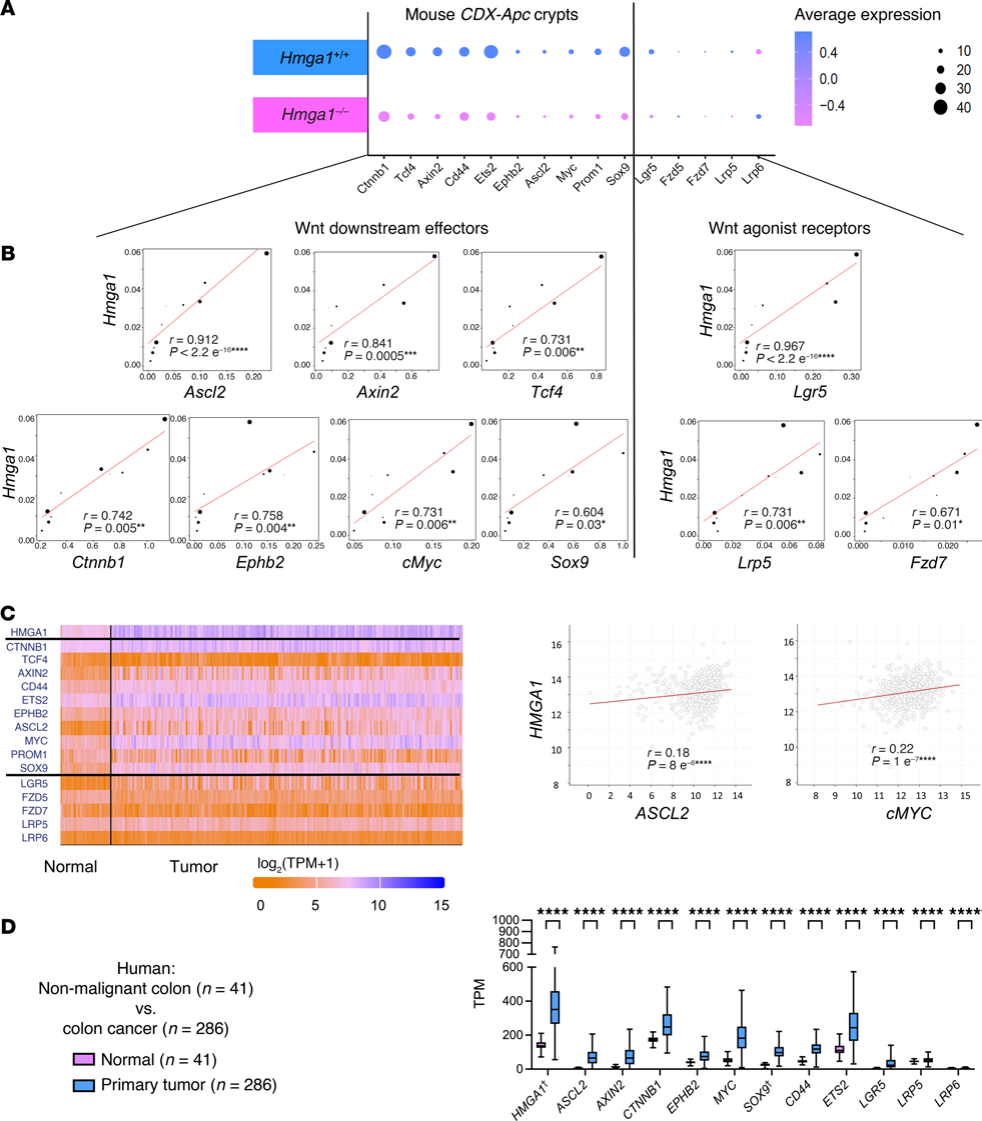
*HMGA1* amplifies Wnt genes in *Apc*-deficient colon crypts, and these HMGA1-Wnt pathways are activated in human colon cancer. (**A**) Dot plot of Wnt effector and receptor gene expression in crypt cells of *CDX2P-CreER^T2^ Apc^fl/fl^* mice with *Hmga1*^+/+^ versus *Hmga1^–/–^*, demonstrating that HMGA1 activates all Wnt effectors and many Wnt receptor genes. (**B**) *Hmga1* is positively and strongly correlated with Wnt genes, including Wnt effector genes (*Ascl2*, *Axin2, Tcf4, Ctnnb1*, *Myc,* *Ephb2,* *Sox9*) and Wnt receptor genes (*Lgr5*, *Lrp5, Fzd7*) (Spearman’s rank correlation test). (**C**) Heatmap showing *HMGA1* and WNT genes. *HMGA1* correlates positively with *ASCL2* and *MYC* (log scale) in human colon cancer. (**D**) *HMGA1* and WNT genes in nonmalignant colon epithelium (*n* = 41) and human colorectal adenocarcinoma (*n* = 286) from TCGA (student’s *t* test). ^†^*HMGA1* and *SOC9* expression were previously reported in ref. 30. TPM, transcripts per million. *P < 0.05, **P < 0.01, ***P < 0.001, ****P < 0.0001.

Given the fundamental role for Wnt signaling in colon tumorigenesis and HMGA1-dependent upregulation of Wnt genes and β-catenin levels in our tumor models, we further examined the relationship between HMGA1 and Wnt genes. We focused on canonical Wnt pathway genes, including Wnt effectors (*Ctnnb1, Tcf4, Axin2, Cd44, Ets2, Ephb2, Ascl2, cMyc, Prom1, Sox9*) and Wnt receptors (*Lgr5, Lrp5, Lrp6, Fzd5, Fzd7*). Remarkably, all Wnt effector genes were upregulated at the level of single cells in the setting of intact HMGA1 and *Apc* deficiency (Figure 11A). Of the Wnt receptors, both *Lgr5* and *Lrp5* transcripts are upregulated in crypt cells with intact HMGA1. We also found significant positive correlations between *Hmga1* and multiple Wnt effectors (Figure 11B) with the strongest correlations (r > 0.68; *P* < 0.05) for *Ascl2, Axin2, Tcf4, Ctnnb1*, *Ephb2*, and *cMyc*. To determine whether HMGA1 enhances chromatin accessibility at promoter regions for these genes, we examined our ATAC-seq results, which revealed increased chromatin accessibility at promoter regions for *Ascl2, Tcf4, Prom1*, *Lgr5,* and *Lrp5* (Supplemental Figure 8). Intriguingly, HMGA1 is also associated with accessible chromatin at the *Hmga1* promoter (Supplemental Figure 8), suggesting that high levels of HMGA1 induce its own expression in *Apc*-deficient crypts by opening chromatin at its promoter region. Together, these results demonstrate that HMGA1 enhances chromatin accessibility to activate Wnt agonist receptor signaling and Wnt effector genes. In small intestinal epithelium, ASCL2 activates Wnt genes (95–98), and upregulation in *Ascl2* could trigger a feed-forward loop whereby HMGA1 activates *Ascl2*, which, in turn, amplifies Wnt gene expression in colon epithelium with Apc inactivation.

### HMGA1 and ASCL2 are upregulated and coexpressed in human colorectal cancer.

To determine which HMGA1 pathways are relevant to human colon tumorigenesis, we queried the Cancer Genome Atlas (TCGA) for expression of HMGA1 and Wnt genes (Figure 11, C and D). As we previously reported, *HMGA1* and *SOX9* are upregulated in colon cancer compared with nonmalignant epithelium (4, 30, 62). Strikingly, most of the Wnt genes upregulated by HMGA1 in our murine model are also upregulated in human colon cancer, including the WNT effectors, *ASCL2, AXIN2, CTNNB1, MYC, EPHB2, CD44,* and *ETS2* and the WNT receptors, *LGR5, LRP5,* and *LRP6* (Figure 11, C and D). Further, both *ASCL2* and *cMYC* are upregulated and positively correlated with *HMGA1,* suggesting that HMGA1 may directly induce their expression in human colon tumorigenesis (Figure 11C).

### HMGA1 upregulates ASCL2 and promotes oncogenic properties in human colon cancer cells.

**Figure 12:**
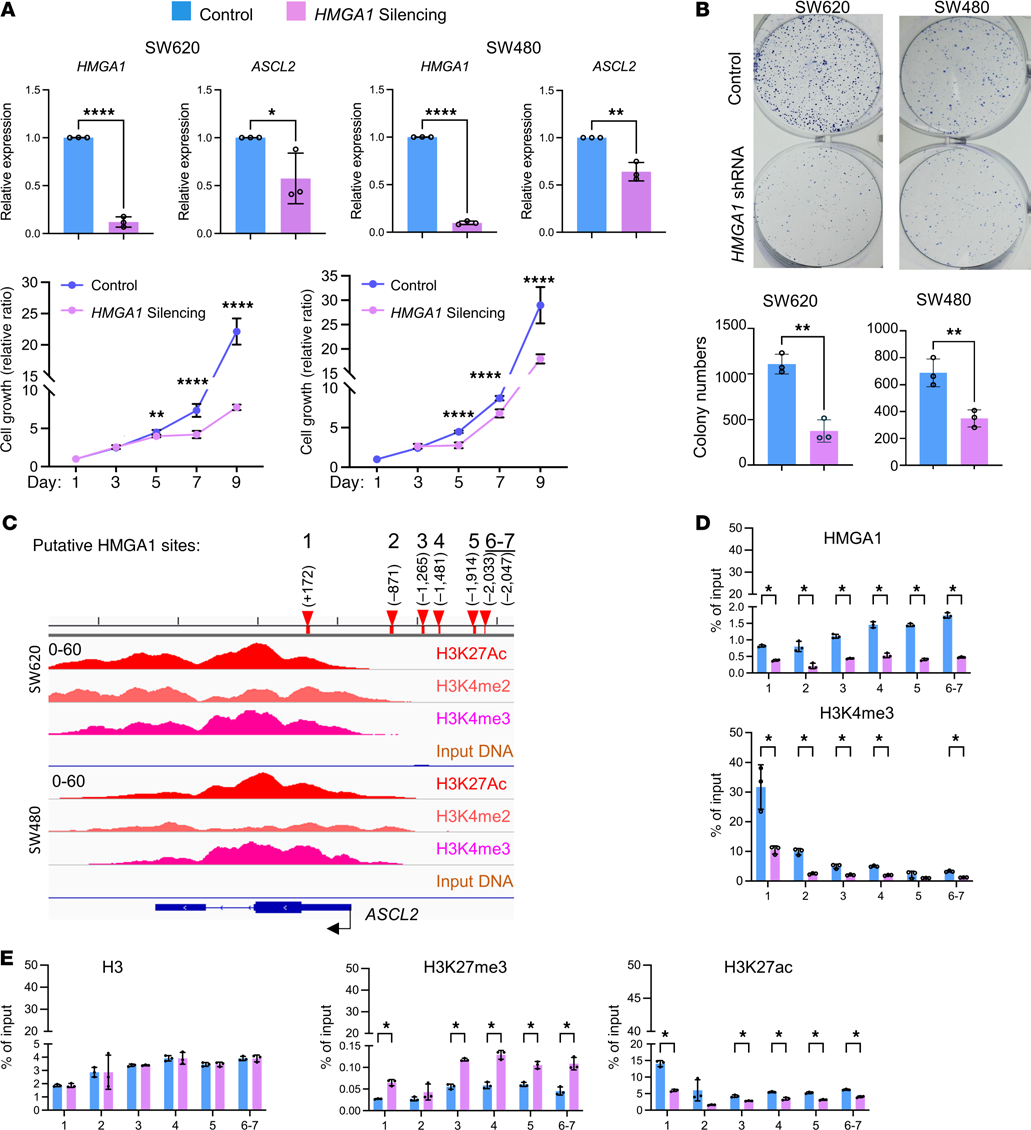
HMGA1 induces *ASCL2* by directly binding to the promoter and recruiting activating histone marks. (**A**) Silencing *HMGA1* represses *ASCL2* and decreases proliferation in SW620 and SW480 cells. Control cells were transduced with empty lentiviral vector versus shRNA targeting *HMGA1*. (*P < 0.05*,* **P < 0.01, ***P < 0.001, ****P < 0.0001; student’s *t* test). (**B**) Silencing *HMGA1* decreases clonogenicity in SW620 and SW480 (**P < 0.01, ****P < 0.0001; student’s *t* test). (**C**) Predicted HMGA1 binding sites 1–5 and region 6–7 in the *ASCL2* promoter region shown with activating histone marks from SW620 and SW480 (GSE106921). (**D**) ChIP assay results at sites 1–5 and region 6–7 in SW620 cells from one representative biological replicate for HMGA1 and activating histone marks (H3K4me3,and H3K27ac). (**E**) ChIP assay results at sites 1–5 and region 6–7 in SW620 cells from one representative biological replicate for H3 and the repressive histone (H3K27me3). (*P < 0.05, **P < 0.01, ***P < 0.001, ****P < 0.0001; student’s *t* test following significance by ANOVA).

The ASCL2 transcription factor is critical to cell fate in the small intestine (95–98) although its role in the colon has not been studied in detail. Because our results strongly link *HMGA1* to *ASCL2* in colon tumorigenesis in humans and mice, we tested whether HMGA1 directly activates *ASCL2* expression in human colon cancer cells. Silencing *HMGA1* in 2 human colon cancer cell lines (SW620, SW480) by lentiviral-mediated delivery of short hairpin RNA (shRNA) or CRISPR/Cas9 represses *ASCL2*, demonstrating that *ASCL2* expression depends on HMGA1 (Figure 12A and Supplemental Figure 9A). Next, we tested whether HMGA1 deficiency affects oncogenic properties in these cells. We previously reported that HMGA1 knockdown decreases clonogenicity in SW480 cells using plasmid-mediated gene silencing (35). Here, we found that *HMGA1* silencing (via shRNA or CRISPR) disrupts proliferation and clonogenicity similarly in SW620 and SW480 cells (Figure 12, A and B, and Supplemental Figure 9, A and B), demonstrating that these in vitro, oncogenic phenotypes depend on high levels of HMGA1. To determine whether *ASCL2* restoration will rescue these phenotypes, we reexpressed *ASCL2* in both colon cancer cell lines (SW620 and SW480) with *HMGA1* silencing (Supplemental Figure 9C). Restoration in *ASCL2* levels results in a partial rescue of proliferation and full rescue of clonogenicity in both cell lines, suggesting that ASCL2 mediates some, but not all, effects of HMGA1 in these colon cancer cells (Supplemental Figure 9, C and D).

### HMGA1 directly induces ASCL2 by binding to its promoter and recruiting activating histone marks in human colon cancer cells.

To ascertain whether HMGA1 binds directly to the *ASCL2* promoter to activate its expression, we used an in silico prediction algorithm (TRAP) (99), which identified 7 potential HMGA1 binding sites within the *ASCL2* promoter-enhancer region (labeled 1, 2, 3, 4, 5, 6, and 7; Supplemental Figure 10A). Because sites 6 and 7 are within 10 base pairs of each other, they could not be resolved by ChIP-PCR, and were therefore denoted region 6–7. Intriguingly, all of these sites are positioned near the homologous regions of HMGA1-dependent accessible chromatin in the mouse *Ascl2* promoter (Figure 12C). By chromatin immunoprecipitation-PCR (ChIP-PCR), we assessed HMGA1 chromatin occupancy at these sites in SW620 cells, as these cells have higher levels of both *HMGA1* and *ASCL2* compared with SW480 cells. There was robust HMGA1 occupancy throughout the *ASCL2* promoter compared with the IgG antibody as a negative control (Supplemental Figure 10, A and B). Next, we compared HMGA1 chromatin binding in SW620 cells with or without *HMGA1* silencing, which showed enrichment for HMGA1 occupancy at the same regions in control cells and depletion of HMGA1 with *HMGA1* silencing, validating the specificity of our HMGA1 antibody (Figure 12D and Supplemental Figure 10C). Because HMGA1 recruits active histone marks to upregulate developmental genes in other settings (28, 29), we tested whether HMGA1 binding associates with activating histones, including histone H3 lysine 4 trimethylation (H3K4me3) and histone H3 lysine 27 acetylation (H3K27Ac), which mark active promoters and enhancers, respectively. These marks also associate with HMGA1 chromatin binding in other tumor settings (28, 29). We found enrichment for both activating histone marks (H3K4me3, H3K27Ac) in the regions of HMGA1 binding in SW620 and SW480 cells from public databases (GSE10692) (Figure 12, C and D). By ChIP-PCR, we found that both H3K4me3 and H3K27Ac bind to the *ASCL2* promoter, with greatest enrichment near the HMGA1 binding site number 1 (located near the transcription start site [TSS]) and these active marks are depleted with *HMGA1* silencing (Figure 12D and Supplemental Figure 10C). By contrast, there was no change with *HMGA1* silencing in the positive control, histone H3, which is a ubiquitous histone that does not modulate gene expression (Figure 12E and Supplemental Figure 10C). We also tested whether HMGA1 depletion enables repressive histones to bind to the *ASCL2* promoter as a mechanism of downregulating *ASCL2* with *HMGA1* silencing. Because the repressive histone 3 lysine 27 trimethyl (H3K27me3) was identified in a colon cancer cell line (HCT116) from a public database (GSE171817), we assessed its binding relative to that of HMGA1. In control SW620 cells with high HMGA1, there was minimal binding of the repressive mark, H3K27me3; however, HMGA1 depletion results in modest, yet significant increases in the H3K27me3 repressive mark at the *ASCL2* promoter enhancer region (Figure 12E and Supplemental Figure 10D).

To determine if HMGA1 activates the *ASCL2* promoter, we cloned the human *ASCL2* promoter sequence (–2.5 kb from the TSS) upstream of the luciferase reporter gene and transfected this construct into colon cancer cell lines (SW620, SW480) (Supplemental Figure 11A). In both cell lines with abundant levels of HMGA1, the *ASCL2* promoter is induced compared with control vector lacking the *ASCL2* promoter sequence (Supplemental Figure 11B). By contrast, *HMGA1* silencing decreases *ASCL2* promoter activity, consistent with HMGA1-dependent activation of the *ASCL2* promoter (Supplemental Figure 11B). Together, our results support a model whereby HMGA1, present in high levels, binds to the *ASCL2* promoter, enhances chromatin accessibility, and recruits activating histones to induce *ASCL2* and downstream Wnt genes, thereby driving tumorigenesis in the setting of *Apc* deficiency. Moreover, our findings further highlight HMGA1 as a promising potential therapeutic target, particularly since loss of HMGA1 in colon epithelium has only subtle effects on epithelial regeneration under homeostatic conditions.

## Discussion

Changes in nuclear structure and function are required for normal development, tissue regeneration, and tumorigenesis (4, 11). While underlying mechanisms remain incompletely understood, chromatin state has emerged as a fundamental player required for diverse cell fate decisions in tumor biology. Embryonic stem cells and tissue-specific, adult stem cells have large nuclei harboring “open” accessible chromatin, which is thought to endow these cells with developmental potency or the capacity to differentiate into diverse progeny with distinct functions (4, 11). Similarly, nuclei in aggressive cancer cells are often enlarged and irregular (4, 11), whereas nuclear compaction accompanies differentiation in normal tissues (4, 11). Though somatic mutations accumulate in adult stem cells over time, particularly in highly proliferative tissues, such as the colon crypts, most mutated cells do not evolve into tumors. Thus, changes in chromatin structure and cell state provide a plausible requisite for tumor development. Indeed, pathologists distinguish cancer cells from nonmalignant cells primarily by alterations in nuclear architecture. These observations suggest that understanding mechanisms underlying chromatin structure and cell state during tumor evolution could reveal strategies to intercept the transition from early neoplasia to invasive cancers. Colon tumorigenesis offers a unique opportunity to study cell state, adult stem cells, and tumorigenesis given the hierarchical organization of stem and progenitors within the crypts along with evidence implicating mutated LGR5+ colon stem cells as a tumor initiating cell (100). Moreover, the incidence of colon cancer is increasing globally, particularly in younger individuals, highlighting the significance of this work (24–26).

Here, we discover that HMGA1 acts as an epigenetic regulator that imposes a stem-like chromatin state within *Apc*-deficient crypt epithelial cells. HMGA1 enhances chromatin accessibility at key loci, leading to activation of gene networks involved in Wnt signaling, proliferation, and inflammation early in tumorigenesis. As an architectural transcription factor, HMGA1 binds to DNA and recruits histones and other chromatin complexes to modulate gene expression, rather than acting on its own. While HMGA1 drives clonal expansion, aberrant differentiation, and transformation in diverse settings, its function in colon tumorigenesis has not been studied in detail despite the fact that it is among the most overexpressed genes in colon cancer compared with nonmalignant epithelium (30, 62). We found that *Hmga1* haploinsufficiency dampens colon tumor development and prolongs survival in 2 models. Importantly, mice with *Hmga1* heterozygosity (and WT *Apc*) have normal development and lifespans (28, 29, 56). By contrast, *Apc* deletion together with intact HMGA1 results in increasing HMGA1 and nuclear β-catenin protein levels, not only at their normal location at the crypt base, but throughout the crypt extending toward the luminal epithelium. Intriguingly, HMGA1 protein levels increase within the colon tumors compared with nontumor colon epithelium, even in the setting of *Hmga1* haploinsufficiency. Precisely how this occurs will require further investigation, although these results underscore the importance of HMGA1 in tumorigenesis in the *CDX2P-CreER^T2^Apc^fl/fl^*. While prior studies show that *HMGA2* is overexpressed in colon cancer and *Hmga2* drives tumorigenesis in mouse models with *Let-7* deficiency (101), we focus on *HMGA1* since transcripts are approximately 100-fold higher than *HMGA2* in colon cancer datasets (TCGA) and in many other human tumors (28–30).

In the Min model following inoculation with ETBF, *Hmga1* haploinsufficiency globally or within the colon epithelium is sufficient to decrease tumorigenesis. Surprisingly, complete loss of *Hmga1* (homozygous deficiency) from colon epithelium decreases tumor incidence similar to that of haploinsufficiency. This was unexpected, since global deletion of *Hmga1* in the biallelic *Apc*-deficient model led to the greatest impact on tumor development and survival. However, in models of pancreatic tumorigenesis (28), we found a similar relationship whereby tissue-specific loss of just one *Hmga1* allele was sufficient to dampen tumorigenesis and prolong survival, akin to results with tissue-specific loss of both *Hmga1* alleles. Intriguingly, crypt depth is similar in the colon with intact *Hmga1* or complete loss of *Hmga1* in the WT *Apc* epithelial compartment. Based on our transcriptomic data showing that HMGA1 deficiency fosters differentiation of stem cells to enterocytes, we surmise that the crypt depth is maintained in the *Hmga1* deficient model lacking *Apc* mutation through skewing of more quiescent stem cells towards proliferating enterocytes. Unfortunately, there are no pharmacologic inhibitors to directly disrupt HMGA1 function in the clinics, although modulating HMGA1 function or levels by approximately 50% is likely to be a more feasible therapeutic goal than a more comprehensive disruption of its function.

To identify potential therapies to disrupt HMGA1 function, we focused on the epigenetic landscape downstream of HMGA1. HMGA1 enhances chromatin accessibility globally, in addition to “opening” regulatory regions of the genome important for activation of proliferation, inflammation, and Wnt signaling genes, including *Ascl2*. ASCL2 functions as a master regulator of stemness in small intestinal epithelium where it activates its own expression and that of downstream Wnt effector and receptor genes (95–97), and in esophageal cancer (98), although its role in colon epithelium had not been studied in detail. A recent study also links *ASCL2* expression to early-onset colorectal cancer in a Japanese cohort (102).

We also found that HMGA1 binds directly to the *ASCL2* promoter region and recruits activating histones (H3K4me3, H3K27Ac) to upregulate its expression. Indeed, *Hmga1* and *Ascl2* are the most tightly coregulated genes in murine crypt epithelium (Figure 7). *Hmga1* is also coregulated with other Wnt effectors (*Axin2, Tcf4, Ctnnb1, cMyc*, *Sox9*) and Wnt agonist receptors (*Lgr5, Lrp6, Fzd7*, and *Ephb2*). In addition, HMGA1 enhances chromatin accessibility at promoter regions for *Tcf4, Prom1*, *Lgr5,* and *Lrp5,* suggesting that it may directly induce these Wnt genes or modify chromatin to facilitate their expression. In small intestinal stem cells and Caco2 cells (a human colon cancer cell line), HMGA1 directly induces *SOX9*, and upregulation of *SOX9* by HMGA1 could enhance expansion in Paneth-like cells, since Paneth cell differentiation in small intestine depends on SOX9 (30). While classical Paneth cells are not present in the colon, Paneth cell metaplasia occurs in the proximal colon in *Apc* mice (73, 74) and in humans with inflammatory bowel disease (103), although their role in tumorigenesis is not yet clear. *HMGA1* also correlates with *MYC* in human colon cancer and prior work in other settings shows that *MYC* directly induces HMGA1 (70). HMGA1 also binds to the *MYC* promoter to induce its expression in embryonic stem cells (34). In colon crypt cells, HMGA1 enhances chromatin accessibility at the *Hmga1* promoter, suggesting that HMGA1 induces its own expression, a feature common to many stemness transcriptional regulators, such as *ASCL2* (95). Given the link between *ASCL2* and early onset colon cancer (102), further studies to explore *HMGA1* and *ASCL2* are warranted. Restoration of *ASCL2* only partially rescues proliferation in colon cancer cell lines with *HMGA1* silencing, indicating that HMGA1 regulates additional networks during colon tumorigenesis.

We also identified inflammatory and proliferative networks that are induced by HMGA1 and associated with HMGA1-dependent accessible chromatin. Both IFN-α and γ signaling networks are upregulated in crypt epithelial cells by HMGA1, leading to activation in IFN-stimulated genes and inflammatory networks, including *signal transduction and activator of transcription 1* and *2* (*Stat1/2*), and chemokine genes encoding C-X-X motif chemokine ligands 9 and 10 (*CXCL9/10*). Importantly, inflammatory cytokines and/or their receptors are often amenable to pharmacologic blockade. Our scRNA-seq results show that tumor-infiltrating T-lymphocytes increase in the setting of HMGA1 deficiency (Figure 7), suggesting that HMGA1 in colon tumor cells may foster an immunologically “cold” tumor microenvironment to facilitate tumor progression. Alternatively, the changes in T cell number could reflect altered transcriptomes from HMGA1 deficiency and associated changes in cell behavior, including proliferation and motility. Given the immune pathways identified from colon epithelial cells, studies focusing on HMGA1 inflammatory networks and immune escape are warranted and could identify new therapeutic strategies.

In summary, we discovered that HMGA1 acts as a molecular key that “opens” chromatin to activate transcriptional networks that maintain a stem and Paneth-like cell state early in colon tumorigenesis. Within crypt epithelial cells, HMGA1 enhances chromatin accessibility to activate the *Ascl2* master regulator gene, additional Wnt genes, and inflammatory networks in murine models with *Apc* inactivation. Further, in human colon cancer, *HMGA1* and *ASCL2* are coexpressed and upregulated along with downstream Wnt pathway genes. Together, our results establish HMGA1 as an epigenetic gatekeeper of ASCL2 and Wnt signals, inflammation, and a stem-like state in colon cells with *APC* inactivation, highlighting HMGA1 as a promising potential therapeutic target in colon cancer.

## Methods

### Sex as a biologic variable.

All studies were carried out on male and female mouse populations and similar findings were observed for both sexes.

Detailed methods, statistical analyses, and reagents are provided in the supplemental material section, including culture medium, primers, antibodies, and in silico approaches (Supplemental Table 5). Sequencing data were deposited into the Gene Expression Omnibus (GSE) with accession numbers GSE279070 (scRNA-seq) and GSE278871 (ATAC-seq).

### Animal models.

*CDX2P-CreER^T2^Apc^fl/fl^* (73, 74) or *Apc^Min/–^* (Min mice) (75–79) mice were previously described. The *CDX2P-CreER^T2^Apc^fl/fl^* were generated and provided in house at the University of Michigan (73, 74). The *Apc^Min/–^* (Min mice) were originally obtained from Bert Vogelstein at Johns Hopkins University who developed this model (104). Both were crossed to mice with global deficiency of one or both *Hmga1* alleles (all on C57Bl6 backgrounds) (28, 29). Tissue-specific *Hmga1*-deficient models were generated by crossing to mice with floxed *Hmga1* alleles. Additional details are provided in the supplement (Supplemental Data Set 1).

### Statistics.

To compare continuous variables across 2 groups, statistical significance was determined using a 2-tailed student’s *t* test when normally distributed (ascertained by Ryan-Joyner and D’Agostino-Pearson tests). If not normal, the Mann-Whitney test was used. To compare more than 2 groups, we used a 1-way ANOVA with Dunnett’s or Turkey’s multiple comparisons (Prism 10, GraphPad Software) after which 2 groups were compared via 2-tailed student’s *t* test if normally distributed or Mann-Whitney if not. For categorical data, association with condition was evaluated by Fisher’s exact test. We compared survival analyses under the assumption of Cox proportional hazards using the log-rank test. *P* < 0.05 was considered significant. All code for the scRNA-seq analysis was performed using Seurat at the indicated resolutions; code will be made available from the corresponding author upon request.

### Study approvals.

All mouse studies were approved by the Johns Hopkins University Institutional Animal Care and Use Committee (IACUC).

### Data availability.

As above, metadata are available in the NCBI GEO database (access numbers: scRNA-seq: GSE279070 and ATAC-seq: GSE278871); the remaining data are provided in the Supporting Data Values file.

## Author contributions

LR and CLS conceptualized the project; LZL, IH, and BEW drafted parts of the manuscript, and LR wrote the final draft, which was reviewed by all authors prior to submission. LZL, JHK, IH, SW, XW, SSP, JC, LC, LX, BEW, JCE, JK, ZT, IM, KLR, YF, ERF, CLS, and LR performed experiments and analyzed data. TL interpreted histology.

## Supplementary Material

Supplemental data

Supplemental data set 1

Unedited blot and gel images

Supplemental table 5

Supporting data values

## Figures and Tables

**Table 1:**
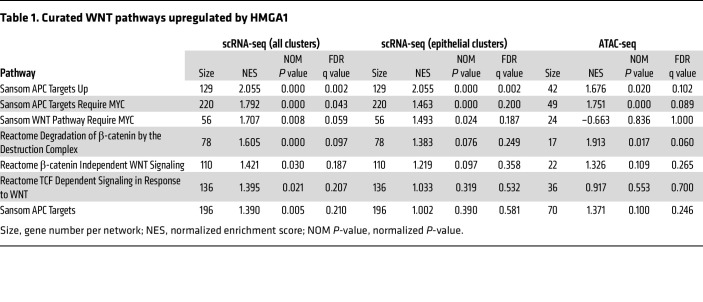
Curated WNT pathways upregulated by HMGA1

## Version 1 02/03/2025

Electronic publication
