# Supplementary material for: HMGA1 acts as an epigenetic gatekeeper of ASCL2 and Wnt signaling during colon tumorigenesis
Source: J Clin Invest. 2025 Feb 3;135(3):e184442. doi: 10.1172/JCI184442 (PMC11785931; doi:10.1172/JCI184442)
Supplement: Supplemental data [file jci-135-184442-s096.pdf]

Supplemental Materials and Methods

Cell lines: SW620 and SW480 human colon cancer cells (American Type Culture Collection, ATCC) were cultured as recommended with STR validation and mycoplasma testing performed on frozen stocks prior to experiments.

Genetic mouse models: *CDX2P-CreER^{T2}Apc$^{fl/fl}$* (1) or *Apc$^{Min/-}$* (Min mice) (2) mice were previously described and crossed to mice with global deficiency of one or both *Hmga1* alleles (C57Bl6 backgrounds) (3, 4). *CDX2P-CreER^{T2}Apc$^{fl/fl}$* models were induced with tamoxifen at 12 weeks of age as described (1). Min mice were inoculated with enterotoxigenic *Bacteroides fragilis* (ETBF) at 5-6 weeks of age as described (3, 4). To generate an intestinal epithelial-specific deletion of *Hmga1*, we crossed *Apc$^{Min/-}$* mice (Min mice) to *Vil-cre*/*Hmga1$^{fl/fl}$* (3) onto C57Bl6 backgrounds (>5 generations) to generate *Vil-cre Apc^{Min} /Hmga1$^{fl/fl}$* and *Vil-cre Apc^{Min} /Hmga1$^{fl/+}$*. Both sexes were used, and mice were sacrificed either at 11-12 weeks for tumor enumeration, or when they appeared ill for survival studies (hunching, decreased activity, decreased oral intake, or loss of > 20% of weight). Tumors in Min mice were enumerated using methylene blue as previously described (2) and normalized across each experiment to the mean tumor count (considered 100%) in Min mice with intact *Hmga1* following inoculation with ETBF. Stools were monitored every 1-2 weeks to ensure that mice remained colonized with ETBF. Tissues were fixed in formalin before processing for histopathology. Weight measurements were normalized to the initial weight to show relative weight gain and account for higher weights in males. Mice were housed in sterile, pathogen-free environments with free access to food and water. Genotypes of all mice were ascertained using primers that were previously published (1-4) in our laboratories or by Transnetyx, Inc.

Lentivirus mediated short hairpin RNA, CRISPR/Cas9 silencing, and overexpression studies in cell lines: Lentiviral delivery of plasmids encoding shRNA targeting *HMGA1* (sh*HMGA1* 1: TRCN0000018949) (RNAi Consortium/TRC) or control lentiviral (expressing empty vector control (SHC001, Millipore Sigma) were used for gene silencing as described (3–5). Cells were transduced using polybrene (8 μg/ml, Millipore Sigma) for 24 h at 37°C and selected with puromycin (1 μg/ml SW620 cells and SW480 cells) for seven days. CRISPR/Cas9 to inactivate *HMGA1* or generate control cells were performed as described (4). Human *ASCL2* overexpression lentiviral vectors were purchased from Vectorbuilder (#VB240408-1517mdk). Lentivirus production and transduction protocols were described previously (4).

Proliferation: Proliferation was assessed as previously described (3) using the 3-(4,5-dimethylthiazol-2-yl)-5-(3- carboxymethoxyphenyl)-2-(4-sulfophenyl)-2H-tetrazolium (MTS; Promega) and phenazine methyl sulfate (PMS; Promega) according to manufacturer's instructions. Cell growth was expressed as a ratio compared to day 1.

Clonogenicity: After lentivirus infection, cells ($1x10^4$) were resuspended in complete medium with puromycin and evenly planted in 6-well plates for colony formation assays. Selection medium was changed every 3-4 days. Colonies were fixed with 4% paraformaldehyde (Millipore Sigma), stained by crystal violet (Millipore Sigma) for 15 min at room temperature, and counted after three weeks.

Luciferase assays: Luciferase assays were performed as previously reported (4) except that the *ASCL2* promoter sequence (-2.5 kb from the transcription start site) was tested. *ASCL2* promoter sequence was amplified by PCR of genomic DNA from SW620 cells and subcloned into a LightSwitch Promoter Reporter Vector (RenSP; SwitchGear Genomics) using NheI and HindIII restriction enzymes (NEB, primer sequences: F-hASCL2 promoter [NheI]: GGTGGTGCTAGCGAGGACCAGATTGGGAAGA, R-hASCL2 promoter [HindIII]: ACCACCAAGCTTCGTCCTAGGTCGTCTGGAG), after which sequence was confirmed (Sanger sequencing). Empty vector was used as a control. Cells (SW480, SW620; $5 x10^5$) were transduced (via Lipofectamine 2000; Thermo Fisher) following the manufacturer's instructions with luciferase constructs and pGL4.53 Vector (Firefly; Promega) as the transduction control. Cells were harvested 48 hours after transfection. An optimized Renilla luminescent reporter gene (RenSP) and firefly luciferase activities were determined using Dual-Luciferase Reporter Assay System (Promega) according to the manufacturer's instructions as we previously described (4).

RNA isolation and quantitative reverse transcriptase (RT) PCR analysis (qPCR): Total RNA from cells or tissue was isolated as previously described (3). Expression of selected genes was detected with primers (**Supplemental Table 2**) in human and mouse samples by quantitative PCR (qPCR), and reactions were performed using Power SYBR Green Master Mix (Thermofisher) on QuantStudio 3 (Thermofisher). Relative expression was determined with *HuPO* as a loading control in human cells and *Gapdh* as a loading control

in mouse samples. All qPCR reactions for gene expression were performed in triplicate. Mean gene expression from each triplicate experiment is shown with biologic replicas as indicated in each figure.

Colon Crypt Dissociation and Isolation: Colon crypts were isolated using an established protocol (6). After flushing the colons with phosphate-buffered saline (PBS), the colon was incised longitudinally after which colon epithelium was collected. Sections (~ 2-3 cm) were incubated in EDTA (3 mM) for 1 hour at 4ºC with vigorous shaking, after which remaining tissue was placed in a new tube for consecutive collection. This process was repeated 4-5 times and fractions were observed under a light microscope to monitor for crypt isolation. After isolation, crypt cells were pelleted and passed through a cell strainer (70 µm). After crypt isolation, cells were processed for single cell RNA sequencing (scRNAseq) and assays for transposase-accessible chromatin-sequencing (ATAC-seq) as described next.

Single cell RNA sequencing (scRNA-seq): Crypt cells were fixed in cold methanol for sequencing via the 10x Chromium platform. Cell Ranger was used to process raw sequencing reads, align them to the mouse genome, and assemble into gene expression count matrices. Count data was further normalized in Seurat followed by clustering, and marker identification. Principal components analysis (PCA) followed by Louvain clustering was performed for dimension reduction to separate cells based on transcriptome. Data are depicted by uniform manifold approximation and projection (UMAP) to visualize clusters based on distinct transcriptomes after which cell identify was imputed using published markers (7-9). Pseudotime analysis to plot and compare a continuous trajectory of cell differentiation dynamics was performed (via Monocle 2) with epithelial island cells from each genotype after transcriptomes were normalized to 2000 cells. Dimensionality reduction was performed [Seurat, discriminative dimensionality reduction via learning tree (DDRtree)]. Cell state analyses was conducted (Monocle 2) to compare static differentiation status of single cells. Cell states were defined by the top 200 differentially expressed genes (n=5 states) across genotypes. Cell cycle analysis was inferred (via CellCycleScoring function in Seurat R) which uses cell cycle specific marker genes (cc genes dataset; Seurat). Differential gene expression was performed to compare single cell transcriptomes across genotypes (via MAST in Seurat with default settings). Genes with log2 (FC) >0.25 and *P* value<0.05 after multiple test correction were considered as differentially expressed. Expression profiles of differentially expressed genes (resolution 0.3) in 12 clusters were computed using relevant software packages (**Supplemental Table 3**).

Assay for transposase-accessible chromatin-sequencing (ATAC-seq): ATAC-seq was performed as previously described (4). Briefly, 50,000 crypt cells each from 3 mice of each genotype (*CDX2P-CreER^{T2}Apc$^{fl/fl}$ Hmga1$^{+/+}$* or *CDX2P-CreER^{T2}Apc$^{fl/fl}$ Hmga1$^{-/-}$* cells) were collected and processed prior to analyses (Azenta/Genewiz for HiSeq 2x150 base pair sequencing). After trimming sequencing adapters and low-quality bases (via Trimmomatic 0.38.), reads were aligned to the reference murine genome (mm10) using bowtie2 and filtered (by Samtools 1.9). The MACS2 2.1.2 algorithm was used for peak calling to identify accessible chromatin regions. Counts encompassed by each region of accessible chromatin were compared quantitatively for differential peak analyses (via Diffbind with default parameters) and mapped to the murine genome assembly (mm10; NCBI).

Gene set enrichment analysis (GSEA): Differential gene expression analyses and plots were generated (R analysis with Seurat) using transcriptomes (scRNAseq) or accessible chromatin (Diffbind; ATACseq) followed by comparisons of pre-ranked GSEA (MSigDb) for Hallmark and curated gene sets (10, 11).

TCGA data mining: We queried transcript abundance for *HMGA1* and *WNT* pathway genes from TCGA database through cBioportal (12) and the University of Alabama at Birmingham Cancer data analysis portal (UALCAN) (13). Boxplots and significance were calculated using GraphPad Prism 10.

Chromatin Immunoprecipitation: ChIP methods were previously described (4). TRAP (14) programs were used to predict the binding sites with primers designed to detect promoter regions (**Supplemental Table 2**) and optimized antibodies (**Supplemental Table 4**) (3-5). ChIP-qPCR results are represented as a percentage of input based on mean quantity derived from a standard curve. ChIP-qPCR was performed in triplicate from each chromatin immunoprecipitation experiment; each immunoprecipitation experiment was repeated at least twice.

Immunohistochemistry (IHC): IHC methods have been described previously (3, 5) except for Ki67 staining. Immunostaining for Ki67 was performed on formalin-fixed, paraffin-embedded (FFPE) sections using an automated stainer (Ventana Discovery Ultra autostainer; Roche Diagnostics). Briefly, following dewaxing and rehydration, epitope retrieval was performed at 96°C for 48 minutes (Ventana Ultra CC1 buffer; catalog#

6414575001, Roche Diagnostics). The primary antibody, anti- Ki67 (1:200 dilution; catalog# Ab16667, Abcam), was applied at 36ºC for 60 minutes. Primary antibodies were detected using an anti-rabbit HQ detection system (catalog# 7017936001 and 7017812001, Roche Diagnostics) followed by detection (Chromomap DAB IHC detection kit, catalog # 5266645001; Roche Diagnostics), counterstaining (hematoxylin), dehydration, and mounting (see **Supplemental Table 4** for antibodies**)**. Quantitative comparison of positively staining cells was performed using QuPath (Version: 0.3.2). For quantitative comparisons of colon crypt composition, we selected fields of similar colon areas and quantified crypts with full lengths based on nuclear staining at 20x magnification. A total of 3-10 fields were selected from 3 mice for each condition.

Immunoblotting (IB): Immunoblots were performed as previously described (3-5) with previously optimized antibodies (**Supplemental Table 4)**. All were repeated at least three times with similar results and quantitatively compared (Image Lab 6.1).

Statistical analysis: When comparing continuous variables across 2 groups, statistical significance was determined using a two-tailed student's *t*-test when normally distributed (ascertained by Ryan-Joyner and D'Agostino-Pearson tests). If not normal, the Mann-Whitney test was used. When comparing more than 2 groups, statistical significance was determined using a one-way ANOVA with Dunnett's or Turkey's multiple comparisons (Prism 10, GraphPad Software) after which 2 groups were compared via student's t-test if normally distributed or Mann-Whitney if not. For categorical data, association with condition was evaluated by Fisher's exact test. Survival analyses were performed under the assumption of Cox proportional hazards and evaluated by log-rank test. $P<0.05$ was considered significant.

Study Approvals: All mouse studies were approved by the Johns Hopkins University Institutional Animal Care and Use Committee (IACUC).

Supplemental Table 1

Cellular marker genes used to impute cell identity of cell clusters with distinct transcriptomes

Cell Type		Genes											
Epithelial	Stem	*Gas5*	*Eef1b2*	*Ung*	*Lgr5*	*Msi1*	*Bmi1*	*Ascl2*	*Cd44*	*Mki67*	*Pcna*	*Smoc2*	*Stmn1*
	Paneth-like	*Lyz1*	*Mmp7*	*Retnlb*	*Chil3*	*Reg3g*	*Deta5*	*Sox9*					
	TA	*Ptma*	*Sfn*	*Itngr2*									
	EC	*Krt19*	*Cdkn1a*	*Vil1*	*Cdh1*								
	Goblet	*Agr2*	*Muc2*	*Ccl9*	*Etv5*	*Tff3*							
T cells	Proliferating Cd8 T	*Prf1*	*Gzma*	*Nkg7*	*Klrd1*	*Ctsw*	*Cd8a*	*Mki67*	*Pcna*	*Stmn1*			
	Cd4 T	*Igfbp4*	*Itm2a*	*Cd40lg*	*Icos*	*Cd4*							
	Cd8 T	*Ctsw*	*Cd8a*	*Lag3*	*Eomes*	*Ptger4*	*Havcr2*	*Pdcd1*					
Other	B cell	*Cd19*	*Ms4a1*	*Spib*									
	Macrophage	*Cd68*											
	Macrophage-like	*Ly6e*	*Cd68*										
	Mast	*Cpa3*	*Hdc*										
	Neutrophil	*Fpr1*	*Csf3r*										

Supplemental Table 2. Primers used for gene expression and ChIP studies

Primers for gene expression studies

Primer		Sequence (5' – 3')
Human *HMGA1*	Forward	AGGAAAAGGACGGCACTGAGAA
	Reverse	CCCCGAGGTCTCTTAGGTGTTGG
Human *ASCL2*	Forward	CGTTCCGCCTACTCGTCG
	Reverse	CTGAGGCTCATAGGTCGAGG
HuPO	Forward	CCATTCTATGATCAACGGGTACAA
	Reverse	AGCAAGTGGGAAGGTGTAATCC
Mouse *Hmga1*	Forward	GCTGGTCGGGAGTCAGAAAG
	Reverse	GGTGACTTTCCGGGTCTTGG
Mouse Gapdh	Forward	AGAAGACTGTGGATGGCCCCTC
	Reverse	GATGACTTGCCCACAGCCTT

Primers for ChIP studies

Primer		Sequence (5' – 3')
h*GAPDH* promoter	Forward	CATCTCAGTCGTTCCCAAAGT
	Reverse	TTCCCAGGACTGGACTGT
h*ASCL2* R1 promoter	Forward	GGGCTCCAGACGACCTAG
	Reverse	GCGACGGGGAAAACTGTG
h*ASCL2* R2 promoter	Forward	GTGGAGGGACGGGGAAAG
	Reverse	GGCTTAAGGAGTGGTCGAGA
h*ASCL2* R3 promoter	Forward	TTTCCCGGACAGTGAGGC
	Reverse	AAGAGATGCGGGGAAATGGT
h*ASCL2* R4 promoter	Forward	CGGAACAGGAAAGCAGCTC
	Reverse	GAAGGTGACCAGATGCTCCT
h*ASCL2* R5 promoter	Forward	CTTGACAAGGGGAAGGAGGG
	Reverse	TGGTCACTTGGCAAATCACAG
h*ASCL2* R6 promoter	Forward	CGCTAAAATCCTGGTGGCTC
	Reverse	CTGCAAACTAGGCCTTGGAA

Supplemental Table 3. Main software used for metadata analysis

**Software**	**version**
R	4.4.0
R studio	2024.04.01+748
IGV	2.16.1
Monocle	2
Seurat	5.1.0
GSEA	4.3.2
Prism	10
MACS2	2.1.2
TRAP	3.05

Supplemental Table 4. Antibodies used for immunoblots, ChIP, flow cytometry and immunohistochemistry

Antibodies for Western Blot

Primary Antibody	Clone/Cat #	Company	Dilution
Anti-HMGA1	EPR7839/ab129153	Abcam	1:1000
Anti-GAPDH	G9545	Sigma	1:2000

Secondary Antibody	Clone/Cat #	Company	Dilution
Goat Anti-Rabbit IgG H+L (HRP)	Ab205718	Abcam	1:2000

Antibodies for Immunohistochemistry

Primary Antibody	Clone/Cat #	Company	Dilution
Anti-HMGA1	EPR7839/ab129153	Abcam	1:1000
Anti-β-Catenin	Ab32572	Abcam	1:100
Anti-Ki67	Ab16667	Abcam	1:200

Secondary Antibody	Clone/Cat #	Company	Dilution
Goat Anti-Rabbit IgG H+L (HRP)	Ab205718	Abcam	1:2000

Antibodies for ChIP experiments

Antibody	Clone/Cat #	Company	Amount (μg)
Anti-HMGA1	EPR7839/ab129153	Abcam	1.5
AntiHMGA1a/HMGA1b	ab4078	Abcam	1.5
Anti-H3K4me3	ab8580	Abcam	3
Anti-H3K27Ac	ab4729	Abcam	3
Anti-H3	ab1719	Abcam	6
Rabbit IgG	NI01-100UG	Millipore Sigma	3

Supplemental References

1. Feng Y, et al. Sox9 induction, ectopic Paneth cells, and mitotic spindle axis defects in mouse colon adenomatous epithelium arising from conditional biallelic Apc inactivation. *Am J Pathol*. 2013;183(2):493–503.
2. Oshima M, et al. Loss of Apc heterozygosity and abnormal tissue building in nascent intestinal polyps in mice carrying a truncated Apc gene. *Proc Natl Acad Sci U S A*. 1995;92(10):4482–4486.
3. Chia L, et al. HMGA1 induces FGF19 to drive pancreatic carcinogenesis and stroma formation. *J Clin Invest*. 2023;133(6). https://doi.org/10.1172/JCI151601.
4. Li L, et al. HMGA1 chromatin regulators induce transcriptional networks involved in GATA2 and proliferation during MPN progression. *Blood*. 2022;139(18):2797–2815.
5. Xian L, et al. HMGA1 amplifies Wnt signalling and expands the intestinal stem cell compartment and Paneth cell niche. *Nat Commun*. 2017;8. https://doi.org/10.1038/ncomms15008.
6. Xian L, et al. Genetic Engineering of Primary Mouse Intestinal Organoids Using Magnetic Nanoparticle Transduction Viral Vectors for Frozen Sectioning [Internet]. 2019. https://app.jove.com/t/57040. Accessed June 17, 2024.
7. Murata K, et al. Ascl2-Dependent Cell Dedifferentiation Drives Regeneration of Ablated Intestinal Stem Cells. *Cell Stem Cell*. 2020;26(3):377-390.e6.
8. Herring CA, et al. Unsupervised Trajectory Analysis of Single-Cell RNA-Seq and Imaging Data Reveals Alternative Tuft Cell Origins in the Gut. *Cell Systems*. 2018;6(1):37-51.e9.
9. Yan H, et al. Single-Cell RNA Sequencing for Analyzing the Intestinal Tract in Healthy and Diseased Individuals. *Frontiers in Cell and Developmental Biology*. 2022;10. https://www.frontiersin.org/articles/10.3389/fcell.2022.915654. Accessed December 4, 2023.
10. Subramanian A, et al. Gene set enrichment analysis: A knowledge-based approach for interpreting genome-wide expression profiles. *Proceedings of the National Academy of Sciences*. 2005;102(43):15545–15550.
11. Mootha VK, et al. PGC-1α-responsive genes involved in oxidative phosphorylation are coordinately downregulated in human diabetes. *Nature Genetics*. 2003;34(3):267–273.
12. Cerami E, et al. The cBio Cancer Genomics Portal: An Open Platform for Exploring Multidimensional Cancer Genomics Data. *Cancer Discovery*. 2012;2(5):401–404.
13. Chandrashekar DS, et al. UALCAN: A Portal for Facilitating Tumor Subgroup Gene Expression and Survival Analyses. *Neoplasia*. 2017;19(8):649–658.
14. Thomas-Chollier M et al. Transcription factor binding predictions using TRAP for the analysis of ChIP-seq data and regulatory SNPs. *Nat Protoc*. 2011;6(12):1860-1869.

Supplemental Figure 1

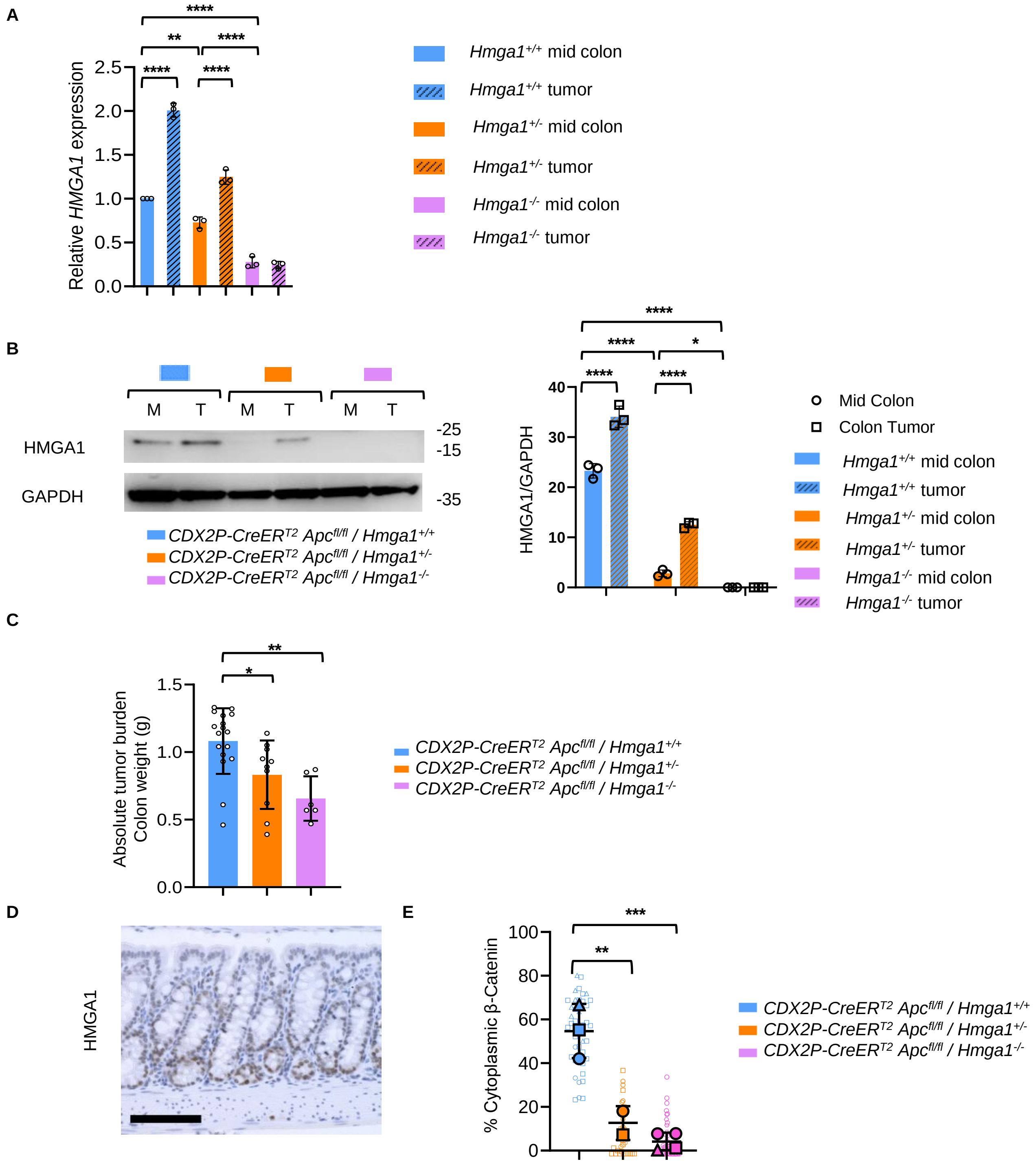

Supplemental Figure 1. HMGA1 levels, tumors, and cytoplasmic β-catenin in Apc-deficient mouse models.

(A) *Hmga1* expression by quantitative PCR (qPCR) in *CDX2P-CreER*^{T2} *Apc*$^{fl/fl}$ with *Hmga1*$^{+/+}$, *Hmga1*$^{+/-}$, or *Hmga1*$^{+/+}$ (****P*<0.001, *****P*<0.0001; Tukey's multiple comparison test following significance by one-way ANOVA). Each bar shows the mean value from qPCR performed in triplicate, the circles represent technical replicates.

(B) Immunoblots showing HMGA1 and control GAPDH in tumor and mid-colon regions of *CDX2P-CreER*^{T2} *Apc*$^{fl/fl}$ models. M: mid colon; T: tumor. Bands from triplicate experiments were compared quantitatively using densitometry (*P*<0.0001; Tukey's multiple comparison test following significance two-way ANOVA).

(**C)** Relative colon weights as a surrogate for tumor burdens in *CDX2P-CreER*^{T2}*/Apc*$^{fl/fl}$ (**P*<0.05 *Hmga1*$^{+/+}$ versus *Hmga1*$^{+/-}$; ***P*<0.005 (Tukey's multiple comparison test following significance by one-way ANOVA).

(D) HMGA1 IHC in colon of wildtype C57Bl6 mice (lacking *Apc* mutation). Bar: 100 µm.

(E) Comparison of cells staining positive for cytoplasmic β-catenin in *CDX2P-CreER*^{T2}*/Apc*$^{fl/fl}$ models (***P*<0.01 *Hmga1*$^{+/+}$ versus *Hmga1*$^{+/-}$; ****P*<0.001 *Hmga1*$^{+/+}$ *versus Hmga1*$^{-/-}$; Tukey's multiple comparison test following significance one-way ANOVA). Each shape corresponds to a different mouse (n=2-4/genotype). The solid shapes show the mean from each mouse; the open, smaller shapes represent individual values/field (range=6-28 fields/mouse) at x20 magnification.

Supplemental Figure 2

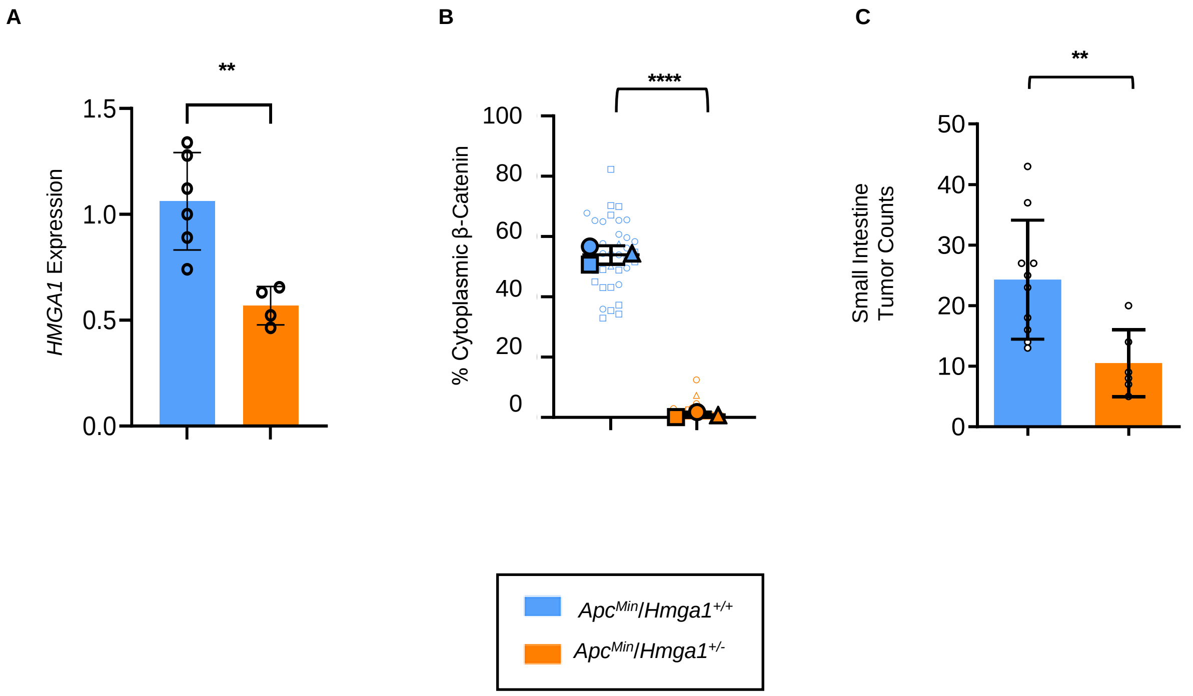

Supplemental Figure 2. *HMGA1* expression, cytoplasmic β-catenin, and small intestinal tumors in Min mouse models.

(A) *Hmga1* expression by qPCR in *Apc*$^{Min/+}$ models with *Hmga1*$^{+/+}$ or *Hmga1*$^{+/-}$ (middle or distal colon; ***P*<0.01; unpaired student's *t*-test). Each circle represents the mean qPCR result performed in triplicate from an individual mouse (n=4-6/genotype)

(B) Comparison of cytoplasmic β-catenin in *Apc*$^{Min/+}$ models at 11-12 weeks post-ETBF (*****P*<0.0001; unpaired student's *t*-test). Each shape corresponds to a different mouse (n=3). The solid shapes show the mean from each mouse; the open, smaller shapes represent individual values/field (range=5-21 fields/mouse) at x20 magnification.

(C) Small intestinal tumors in *Apc*$^{Min/+}$ models at 11-12 weeks post ETBF (***P*<0.01; unpaired student's *t*-test).

Supplemental Figure 3

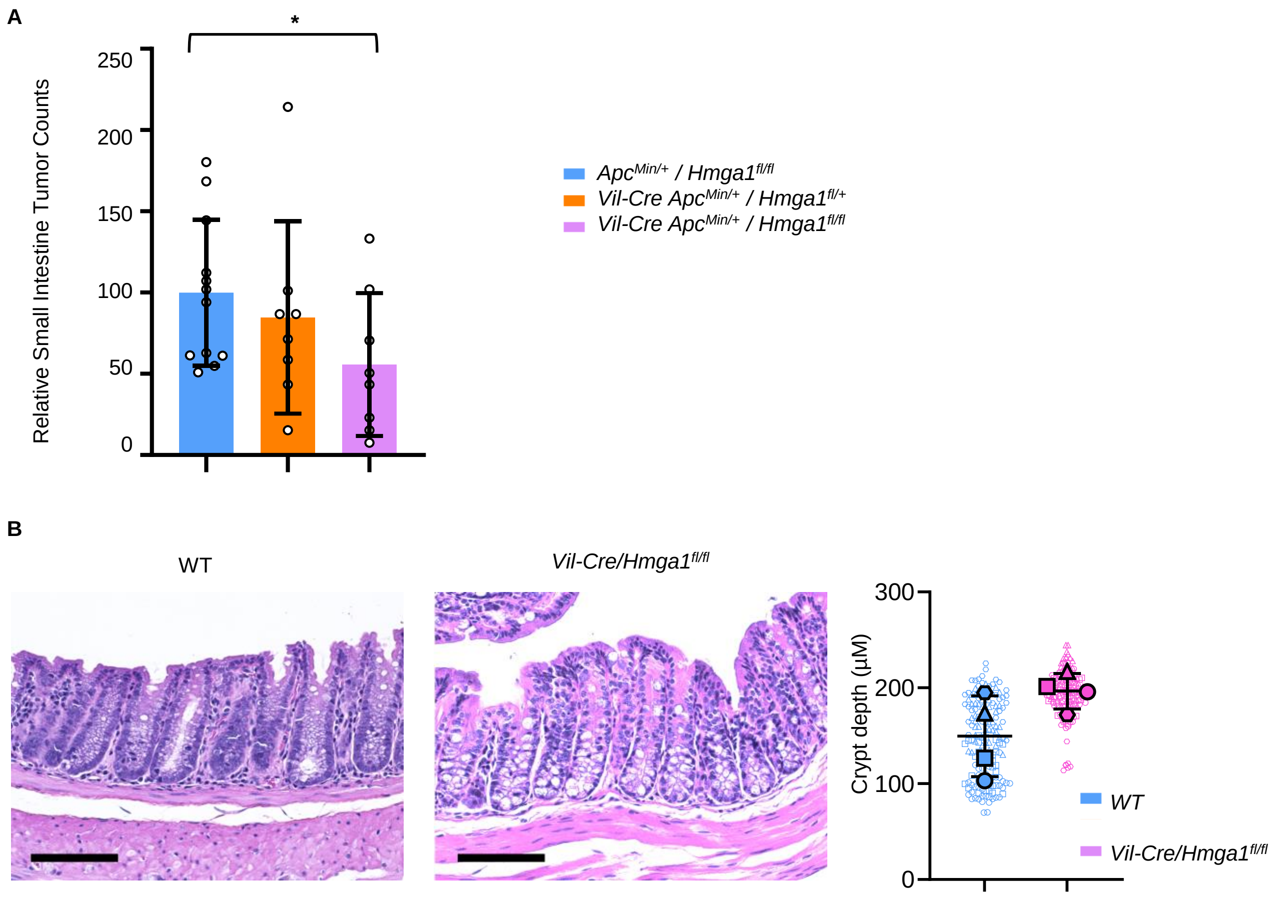

Supplemental Figure 3. Small intestinal tumors in Min models with tissue-specific HMGA1 deficiency and colon histology in wildtype mice with tissue-specific HMGA1 deficiency.

(A) Small intestinal tumor numbers in *Apc*$^{Min/+}$ mice with intact *Hmga1* compared to tissue-specific heterozygous loss of *Hmga1* and tissue-specific homozygous *Hmga1* loss at 11-12 weeks post-ETBF. (*$P<0.05$; Mann-Whitney test). Each dot represents relative tumor number/mouse (n=8-12 mice/genotype).

(B) Representative images (H&E) of colon and crypt depth in WT mice (lacking *Apc* deficiency) with intact *Hmga1* versus WT mice with gut epithelial-specific homozygous *Hmga1* loss. For the graph of crypt depth, the solid shapes show the mean value from each mouse; the open, smaller shapes represent individual measurements/crypt (range=24-53 crypts/mouse) at x20 magnification ($P=0.09$ by unpaired student's *t*-test). Bar: 100 µm.

Supplemental Figure 4

A

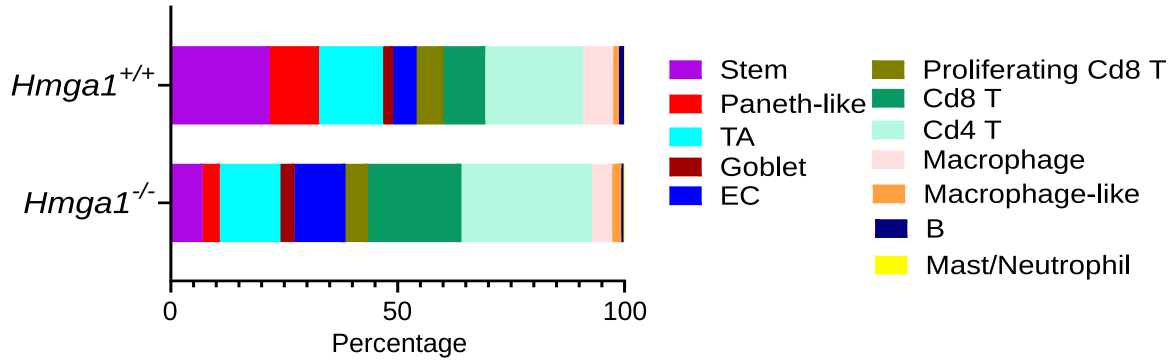

	Hmga1$^{+/+}$	*Hmga1*$^{-/-}$	P value
Stem	21.8	7.1	<0.0001****
Paneth-like	10.9	3.7	<0.0001****
TA	14.1	13.4	0.23
Goblet	2.2	3.1	0.001**
EC	5.2	11.2	<0.0001****
Proliferating Cd8 T	5.9	5.0	0.015*
Cd8 T	9.2	20.6	<0.0001****
Cd4 T	21.5	28.7	<0.0001****
Macrophage	6.7	4.5	<0.0001****
Macrophage-like	1.3	2.0	0.0003***
B	1.1	0.5	<0.0001****
Mast/Neutrophil	0.2	0.4	0.0012**

B

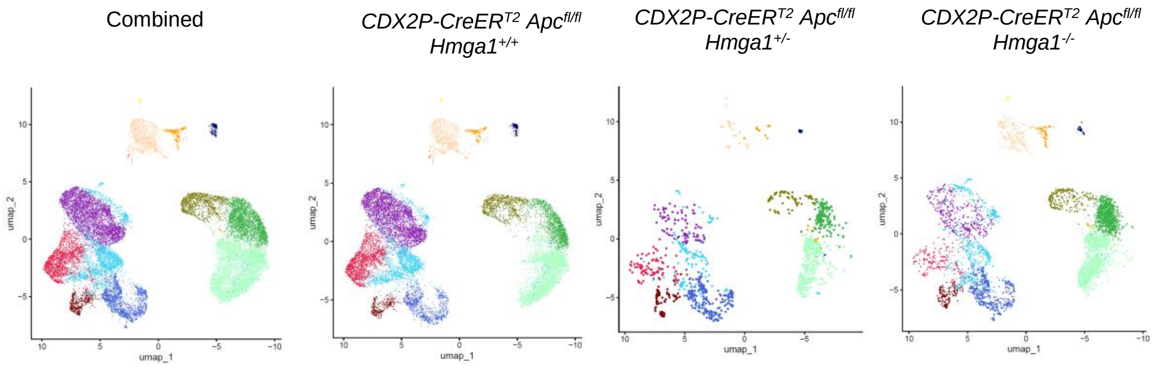

C

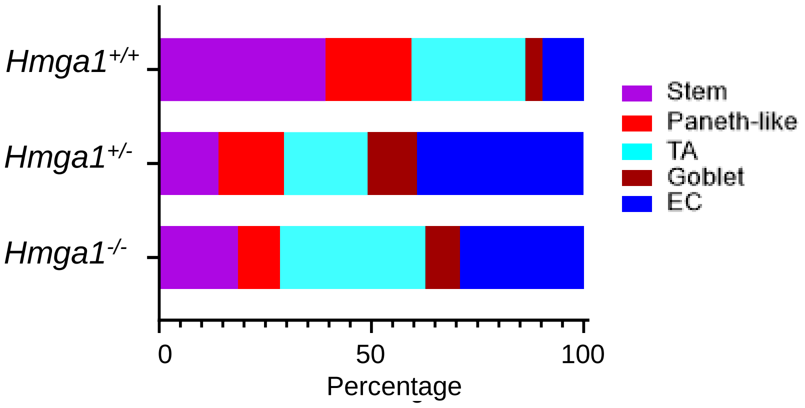

	Hmga1$^{+/+}$	*Hmga1*$^{+/-}$	*Hmga1*$^{-/-}$	*P* value: *Hmga1*$^{+/+}$ versus *Hmga1*$^{-/-}$	*P* value: *Hmga1*$^{+/+}$ versus *Hmga1*$^{+/-}$	*P* value: *Hmga1*$^{+/-}$ versus *Hmga1*$^{-/-}$
**Stem**	39.17	13.99	18.54	<0.0001****	<0.0001****	0.0118*
**Paneth-like**	20.26	15.38	9.89	<0.0001****	0.0047**	0.0002***
**TA**	26.79	19.76	34.28	<0.0001****	0.0002***	<0.0001****
**Goblet**	4.10	11.54	8.13	<0.0001****	<0.0001****	<0.0001****
**EC**	9.68	39.34	29.16	<0.0001****	<0.0001****	0.148 (NS)

Supplemental Figure 4. *Hmga1* deficiency changes composition of colon stem and progenitor cell populations in a dose-dependent manner.

(A) Relative distributions of all cell types identified in the crypt cells from scRNA-seq analysis of *CDX2P-Cre*^{ERT2} *Apc*$^{fl/fl}$ mice with *Hmga1*$^{+/+}$ or *Hmga1*$^{-/-}$ by bar graph (top) or Table (bottom).

(B) UMAP depictions of epithelial island subset from scRNAseq of *CDX2P-CreER*^{T2} *Apc*$^{fl/fl}$ mice with intact *Hmga1*$^{+/+}$, *Hmga1*$^{+/-}$ heterozygosity, *or Hmga1*$^{-/-}$ homozygosity.

(C) Bar graphs (top) and Table (bottom) showing relative distribution of crypt cells by genotype. The association between cell and HMGA1 status was evaluated by chi-squared test for each cell type versus all others.

Supplemental Figure 5

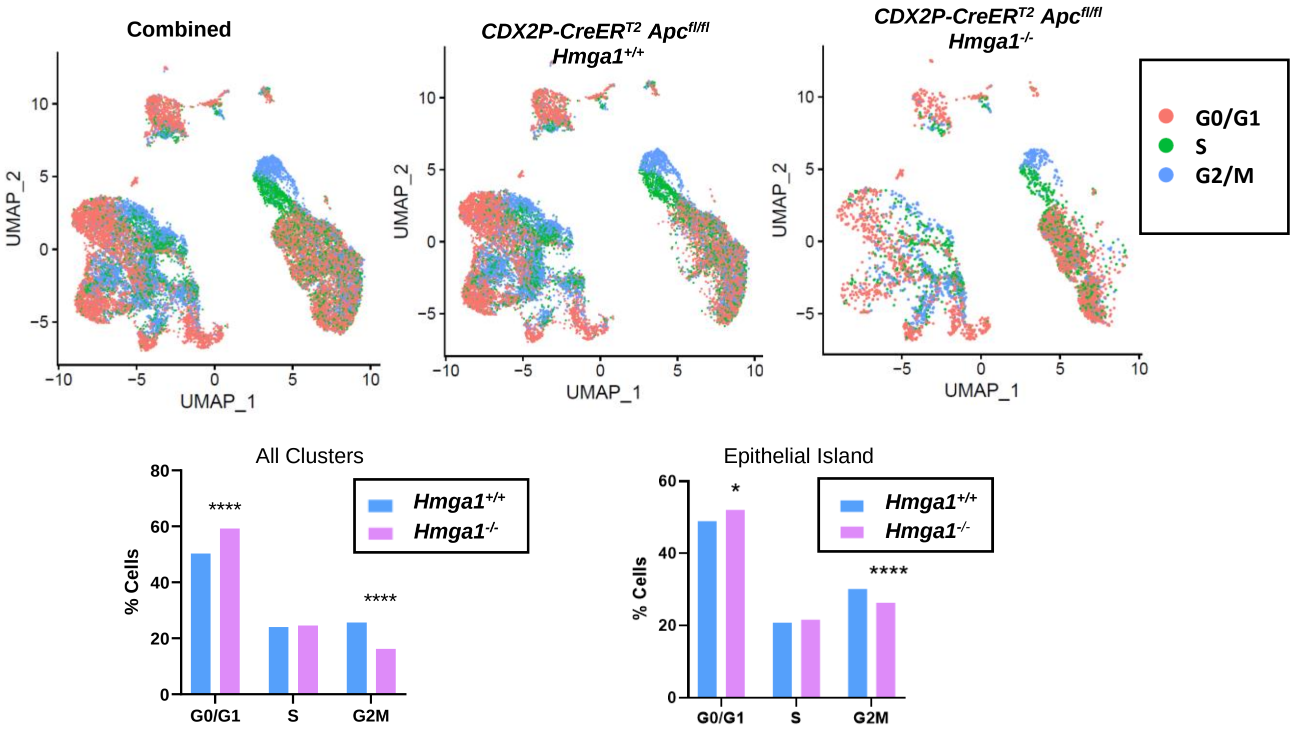

Supplemental Figure 5. Single cell transcriptomes show that HMGA1 increases gene networks involved in cell cycle progression. Cell cycle states inferred from scRNA-seq shown by UMAP (top) or by quantitative comparisons of cell cycle from all clusters (bottom left) or the epithelial island (bottom right) in *CDX2P-CreER*^{T2} *Apc*$^{fl/fl}$ crypt cells with *Hmga1*$^{+/+}$ or *Hmga1*$^{-/-}$. Intact HMGA1 cells have a decrease in the proportion of cells in G0/G1 with an increase in cells reaching G2M (**P*<0.05, *****P*<0.0001; chi-squared) and similar proportions of cells in S phase compared to those with *Hmga1* deficiency.

Supplemental Figure 6

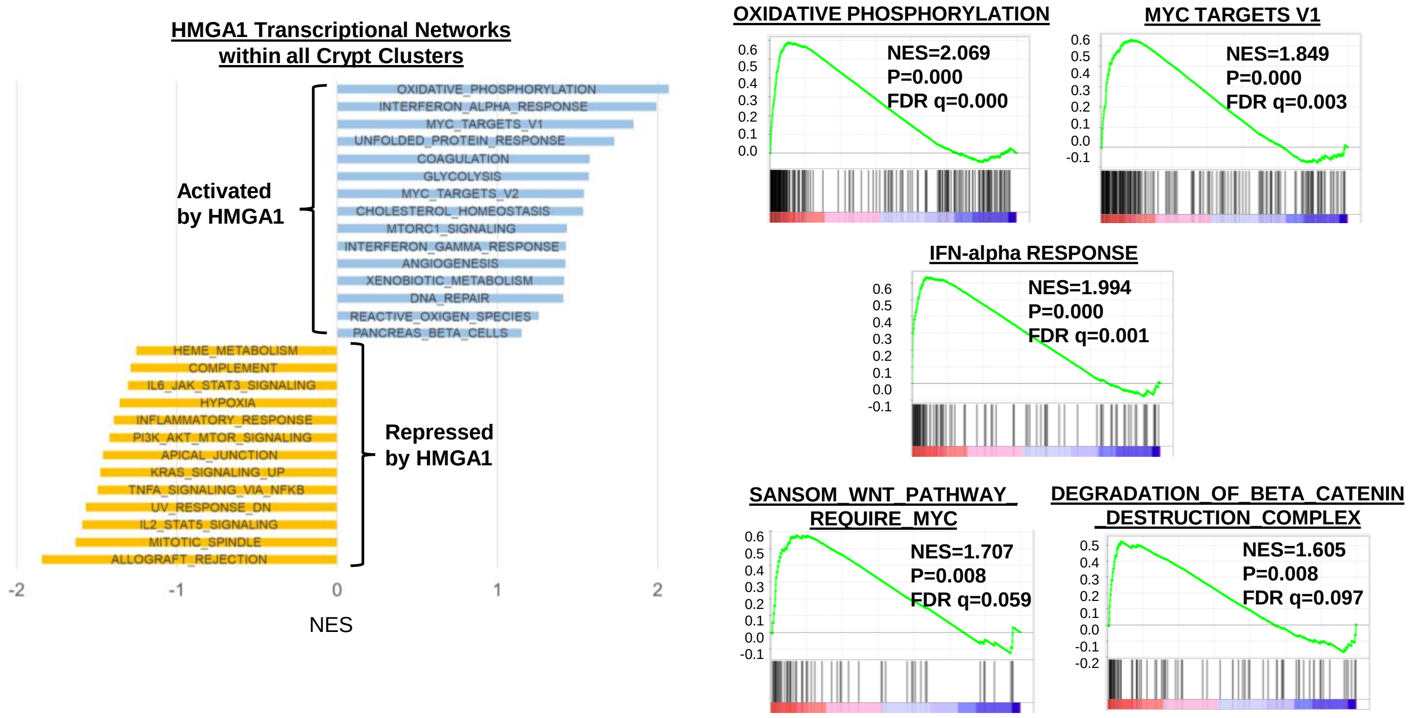

Supplemental Figure 6. HMGA1 activates gene networks in crypt cells involved in metabolism, inflammation, proliferation, and Wnt signaling.
GSEA analysis (left) of single cell transcripts from all clusters combined reveals that HMGA1 activates pathways involved in metabolism (oxidative phosphorylation, glycolysis), proliferation (MYC Targets V1, V2), and inflammation (interferon α, interferon γ) while repressing pathways involved in immune attack (allograft rejection); false discovery rates (FDR)<0.25. Enrichment plots (right) show HMGA1 gene networks in more detail, including genes involved in metabolism and cell cycling (oxidative phosphorylation, MYC), inflammation (interferon alpha), and Wnt signaling. The pathway heterogeneity likely reflects the diverse cell types within the crypts (epithelial and immune cells). Normalized enrichment score (NES) and normalized *P* values are indicated.

Supplemental Figure 7

MYC TARGETS V1

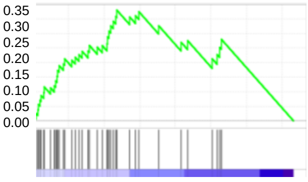

NES=3.380
P=0.000
FDR q=0.000

E2F TARGETS

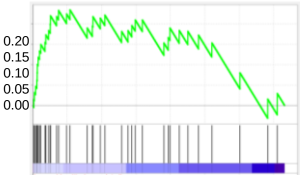

NES=1.894
P=0.000
FDR q=0.048

DEGRADATION_OF_BETA_CATENIN
DESTRUCTION_COMPLEX

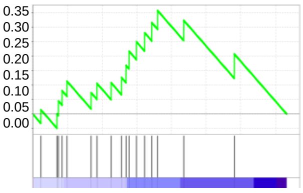

NES= 1.913
P=0.017
FDR q=0.060

Supplemental Figure 7. Single cell transcriptomes show that HMGA1 increases gene networks involved in cell cycle progression. GSEA enrichment plots of curated gene sets demonstrate that HMGA1 enhances chromatin accessibility at gene networks involved in proliferation (MYC, E2F) and Wnt signaling in the epithelial island crypt cells of *CDX2P-CreER*^{T2} *Apc*$^{fl/fl}$ mice.

Supplemental Figure 8

A

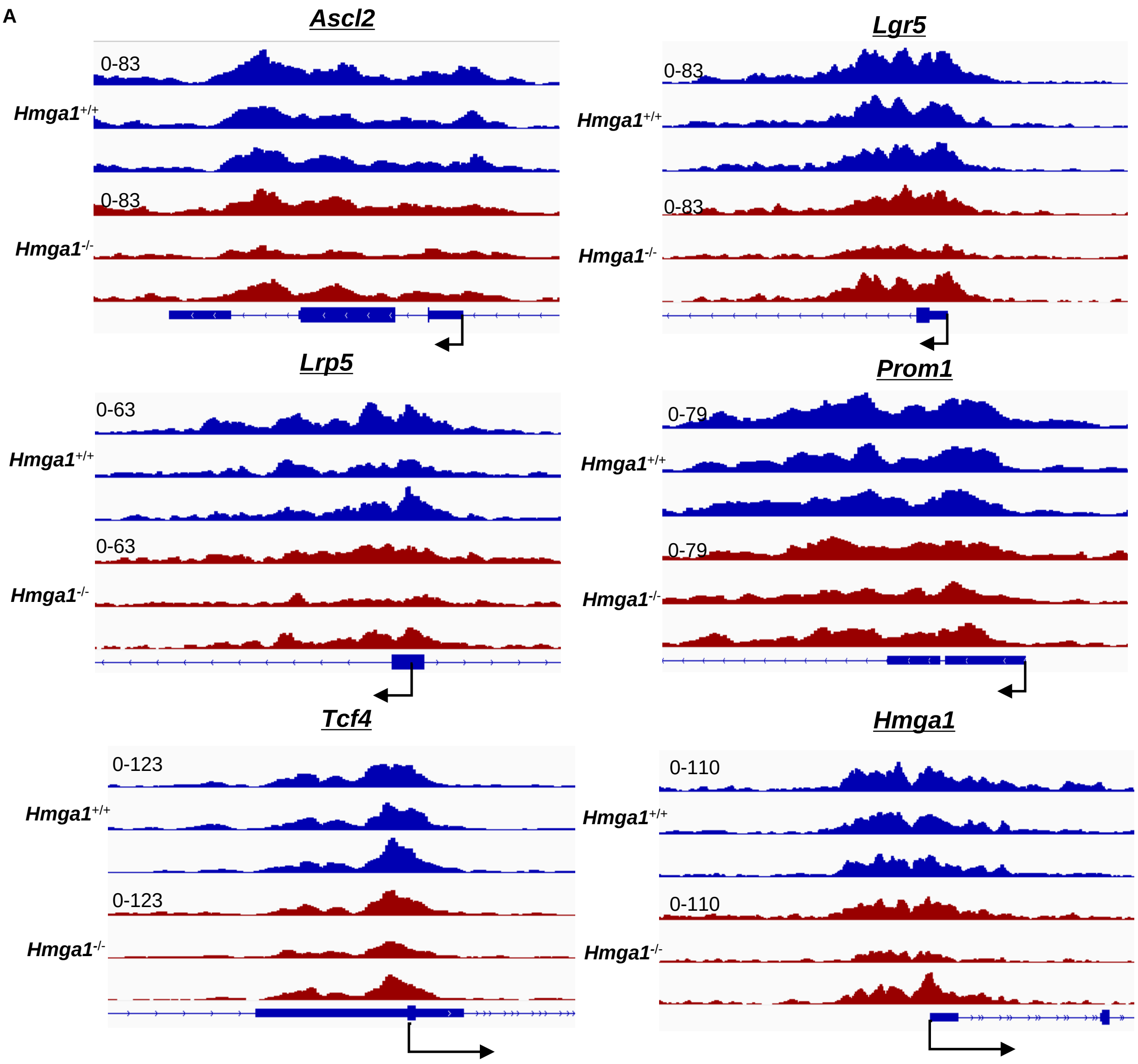

B

Gene	*Ascl2*	*Lgr5*	*Lrp5*	*Prom1*	*Tcf4*	*Hmga1*
Chromosome	chr7	chr10	chr19	chr5	chr18	chr17
Start	142967368	115585991	3685038	44099927	69592438	27555209
End	142968689	115588041	3686928	44102045	69593796	27557008
Peak width (bp)	1322	2051	1891	2119	1359	1800
Log fold change	0.47	0.48	0.48	0.42	0.42	0.41
P value	0.00903	0.00371	0.00719	0.00289	0.00481	0.00835
FDR	0.0437	0.0268	0.0385	0.0232	0.0309	0.0418
Promoter Annotation	Promoter (≤1kb)	Promoter (≤1kb)	Promoter (≤1kb)	Promoter (≤1kb)	Promoter (≤1kb)	Promoter (≤1kb)

Supplemental Figure 8. HMGA1 enhances chromatin accessibility at Wnt loci.
(A) HMGA1 enhances chromatin accessibility at promoter regions for Wnt genes and the *Hmga1* gene in *CDX2P-CreER*^{T2} *Apc*$^{fl/fl}$ crypt cells. ATAC-seq tracks are shown from triplicate samples of each condition.
(B) Table with chromosome number, genome location, peak width in base pairs (bp), log fold change, *P* value, false discovery rate (FDR), and promoter region for HMGA1-dependent, enhanced chromatin accessibility (ascertained from MACS2 calling algorithm).

Supplemental Figure 9

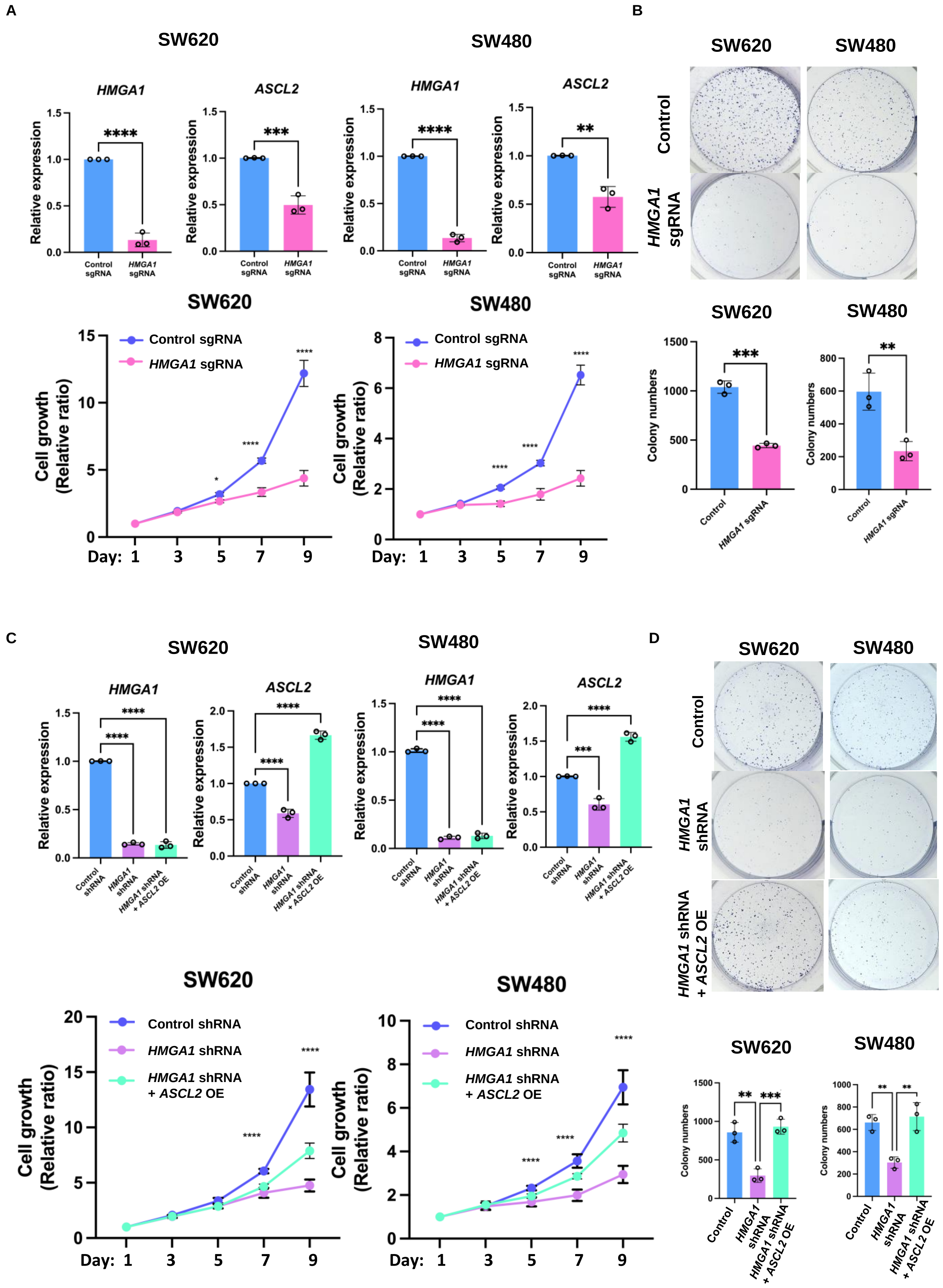

Supplemental Figure 9. HMGA1 up-regulates *ASCL2* in colon cancer cells and re-expressing *ASCL2* rescues proliferation and clonogenicity in HMGA1-deficient colon cancer cells.

(A) Silencing *HMGA1* via CRISPR decreases *ASCL2* expression (top panels) and disrupts proliferation (bottom panels) in human colon cancer cell lines (SW620 left, SW480 right). Each circle represents the mean value from repeat qPCR results performed in triplicate.

(B) Silencing *HMGA1* via CRISPR decreases clonogenicity in human colon cancer cell lines (SW620 left, SW480 right). Each circle represents the mean value from repeat experiments performed in triplicate.

(C) Re-expressing *ASCL2* (top panels, OE=overexpression) in colon cancer cell lines (SW620 left, SW480 right) partially rescues proliferation (bottom panels) in colon cancer cells with *HMGA1* silencing. Each circle represents the mean value from repeat qPCR results performed in triplicate.

(D) Re-expressing *ASCL2* in colon cancer cell lines (SW620 left, SW480 right) rescues clonogenicity in colon cancer cells with HMGA1 silencing. Each circle represents the mean value from repeat experiments performed in triplicate.

(*P<0.05, **P<0.01, ***P<0.001, ****P<0.0001; student's t-test following significance by ANOVA).

Supplemental Figure 10

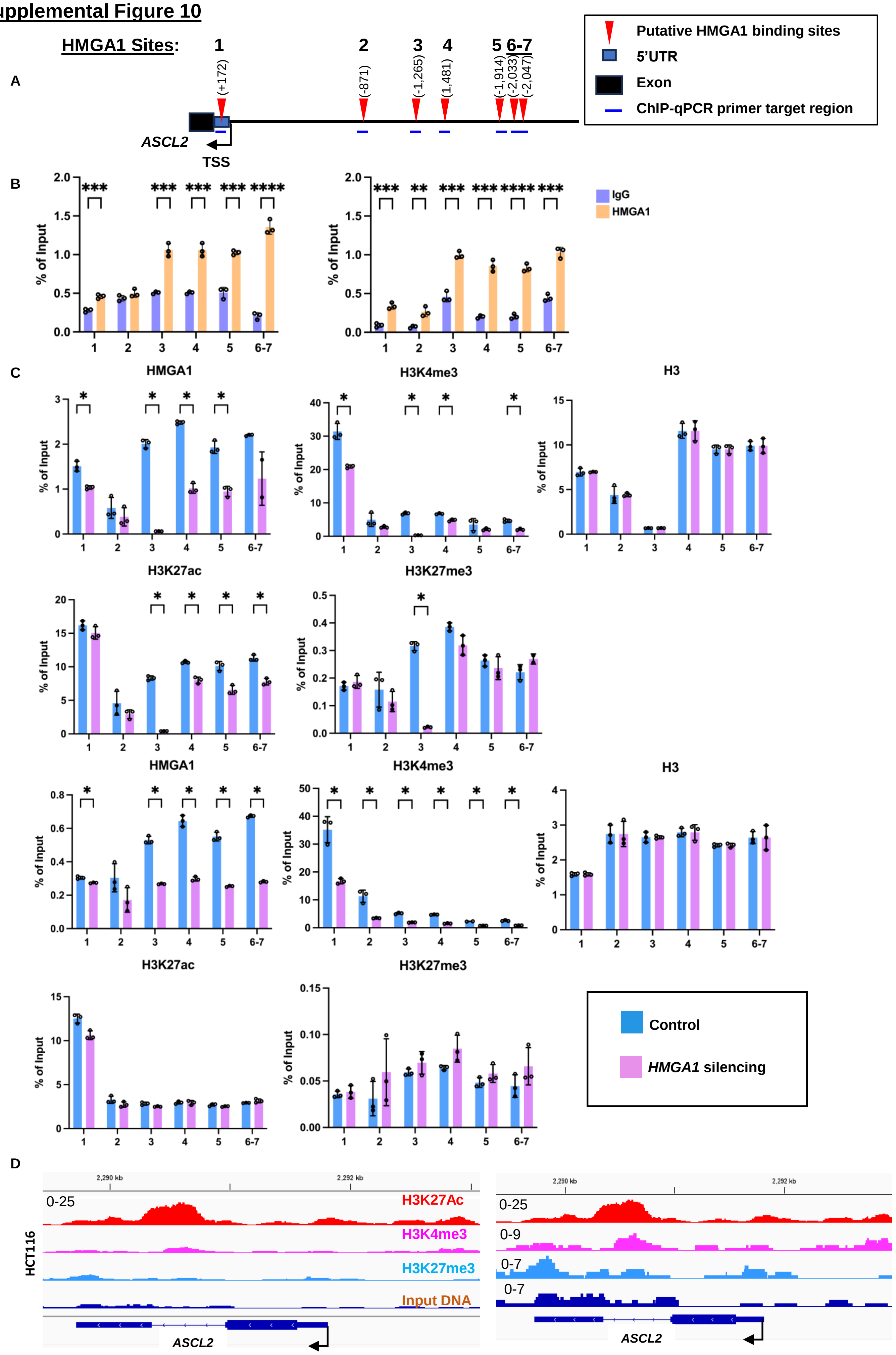

Supplemental Figure 10. HMGA1 occupies the *ASCL2* promoter region and recruits activating histone marks.

(A) *ASCL2* promoter with putative HMGA1 binding sites. TSS: transcription start site.

(B) Enrichment for HMGA1 chromatin occupancy throughout the *ASCL2* promoter region in SW620 cells by ChIP-PCR for HMGA1 compared to ChIP-PCR for IgG as a negative control in repeat chromatin immunoprecipitation experiments (left, right). qPCR was performed in triplicate in each ChIP experiment. (**P*<0.05, student's *t*-test)

(C) ChIP-PCR for HMGA1 occupancy in SW620 cells show similar results in replica ChIP experiments (top, bottom). IgG and H3 ChIP binding controls were compared with *HMGA1* silencing. qPCR was performed in triplicate in each ChIP experiment. (**P*<0.05, student's t-test).

(D) The repressive histone mark, H3K27me3, and activating marks (H3K27ac, H3K4me3) by ChIP-seq in HCT116 cells compared to input DNA (GSE171817) with group autoscaling (left) and individual track autoscaling (right). (**P*<0.05, ***P*<0.01, ****P*<0.001, *****P*<0.0001; student's *t*-test following significance by ANOVA).

Supplemental Figure 11

A

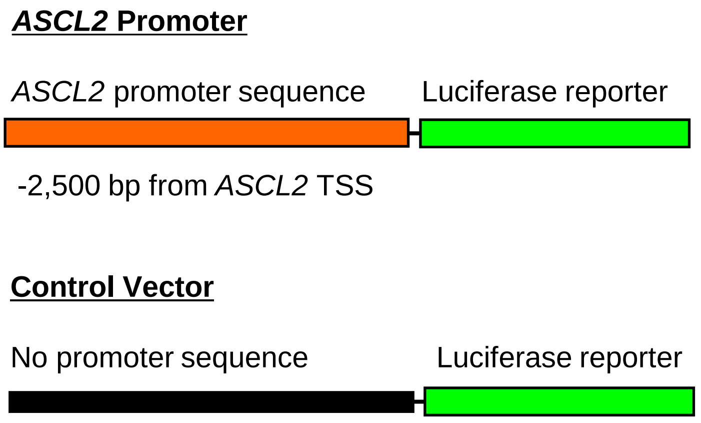

B

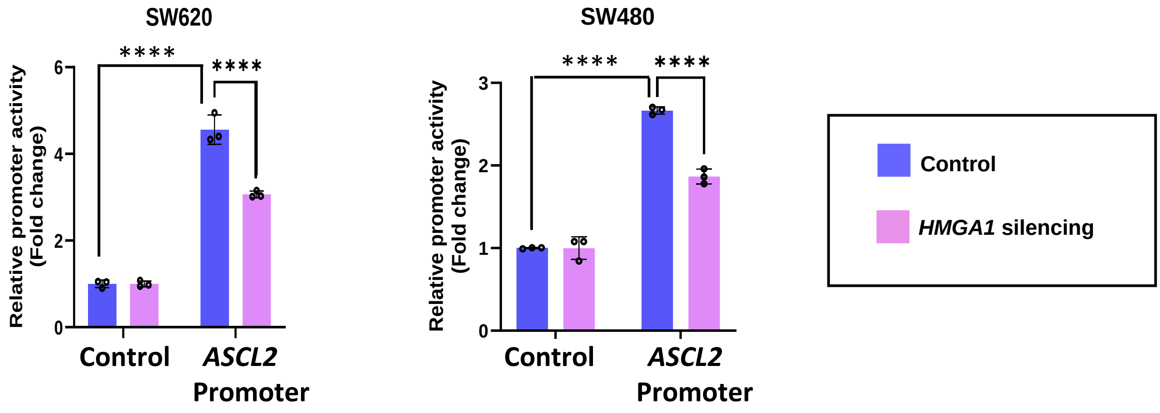

Supplemental Figure 11. HMGA1 transactivates the *ASCL2* promoter.
(A) Reporter constructs transfected into human colon cancer cell lines (SW620, SW480). TSS - transcription start site.
(B) Luciferase assays show activation of the *ASCL2* promoter in colon cancer cells compared to control vector lacking promoter sequence. *HMGA1* silencing decreases promoter activation in both cell lines. (****P<0.0001; two-way ANOVA). Each circle represents the mean value from repeat experiments performed in triplicate.
