# Supplementary material for: HMGA1 acts as an epigenetic gatekeeper of ASCL2 and Wnt signaling during colon tumorigenesis
Source: J Clin Invest. 2025 Feb 3;135(3):e184442. doi: 10.1172/JCI184442 (PMC11785931; doi:10.1172/JCI184442)
Supplement: Unedited blot and gel images [file jci-135-184442-s098.pdf]

Full unedited blot for Supplemental Fig. 1B

Biological replicate 1   2022-11-21

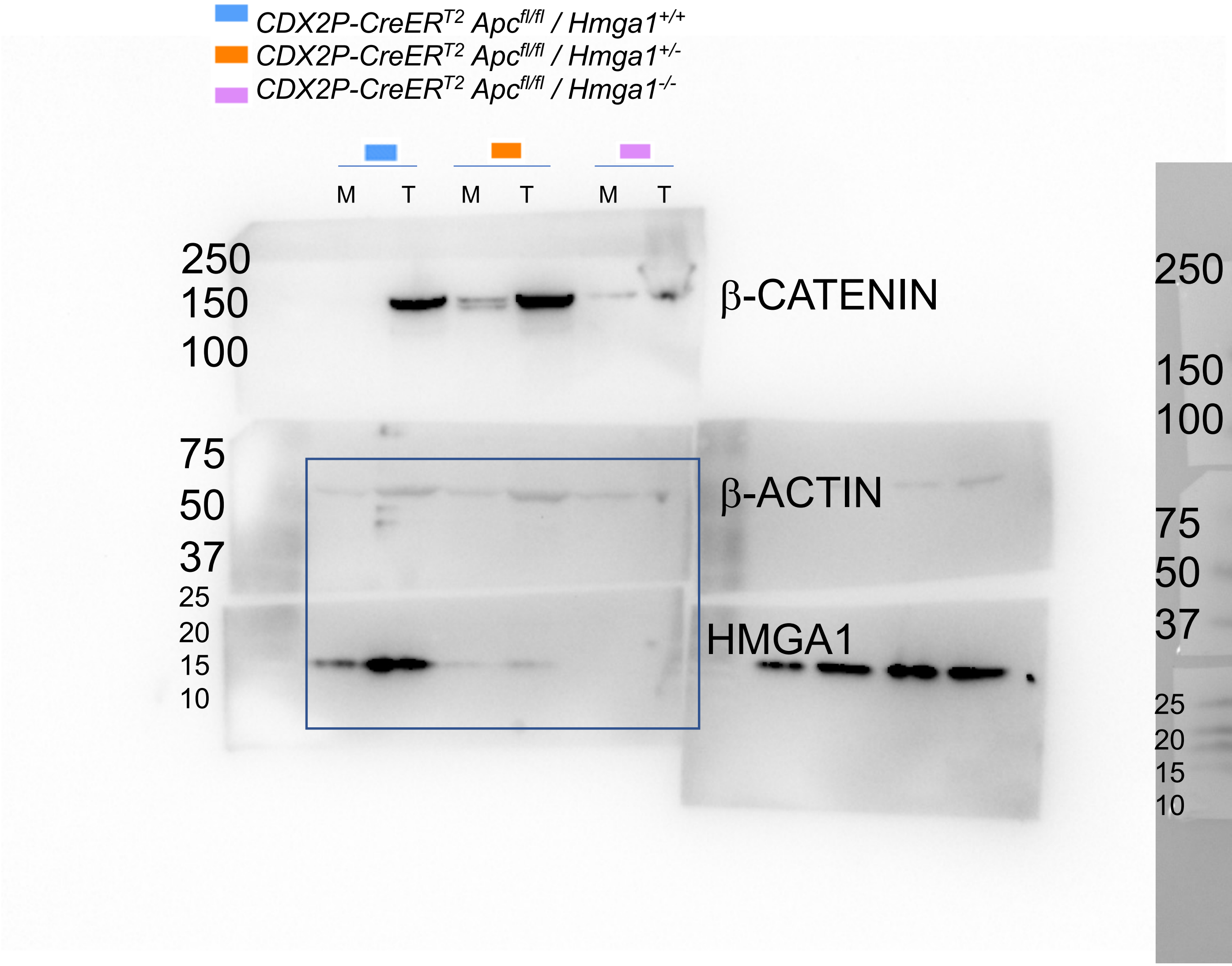

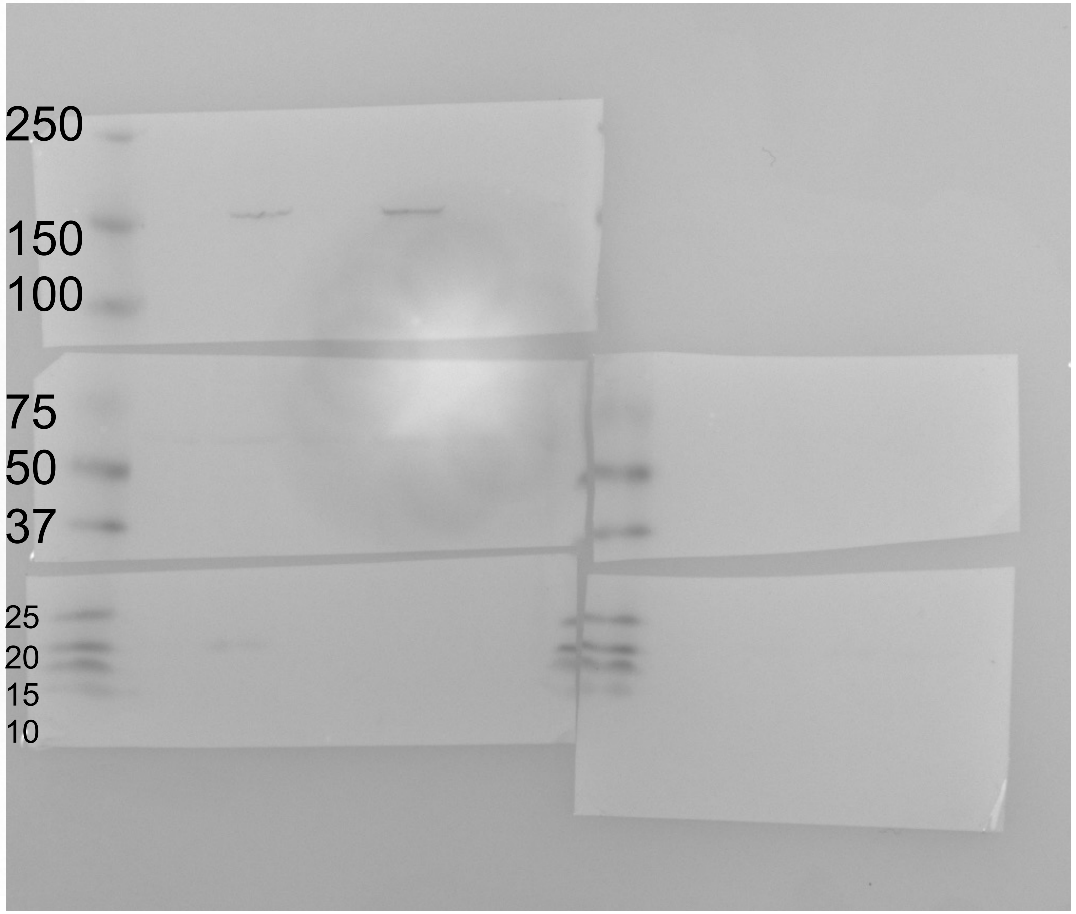

Full unedited blot for Supplemental Fig. 1B **(used for figure)**

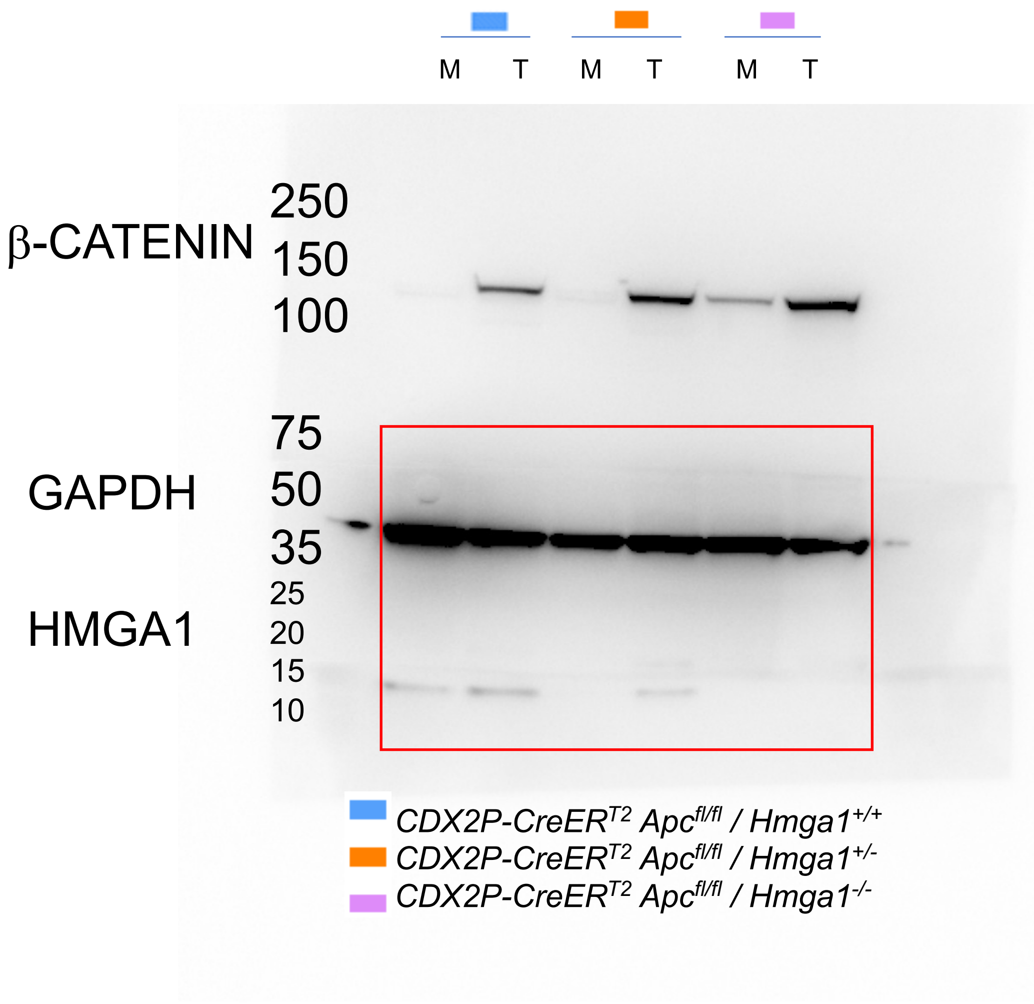

Low exposure time

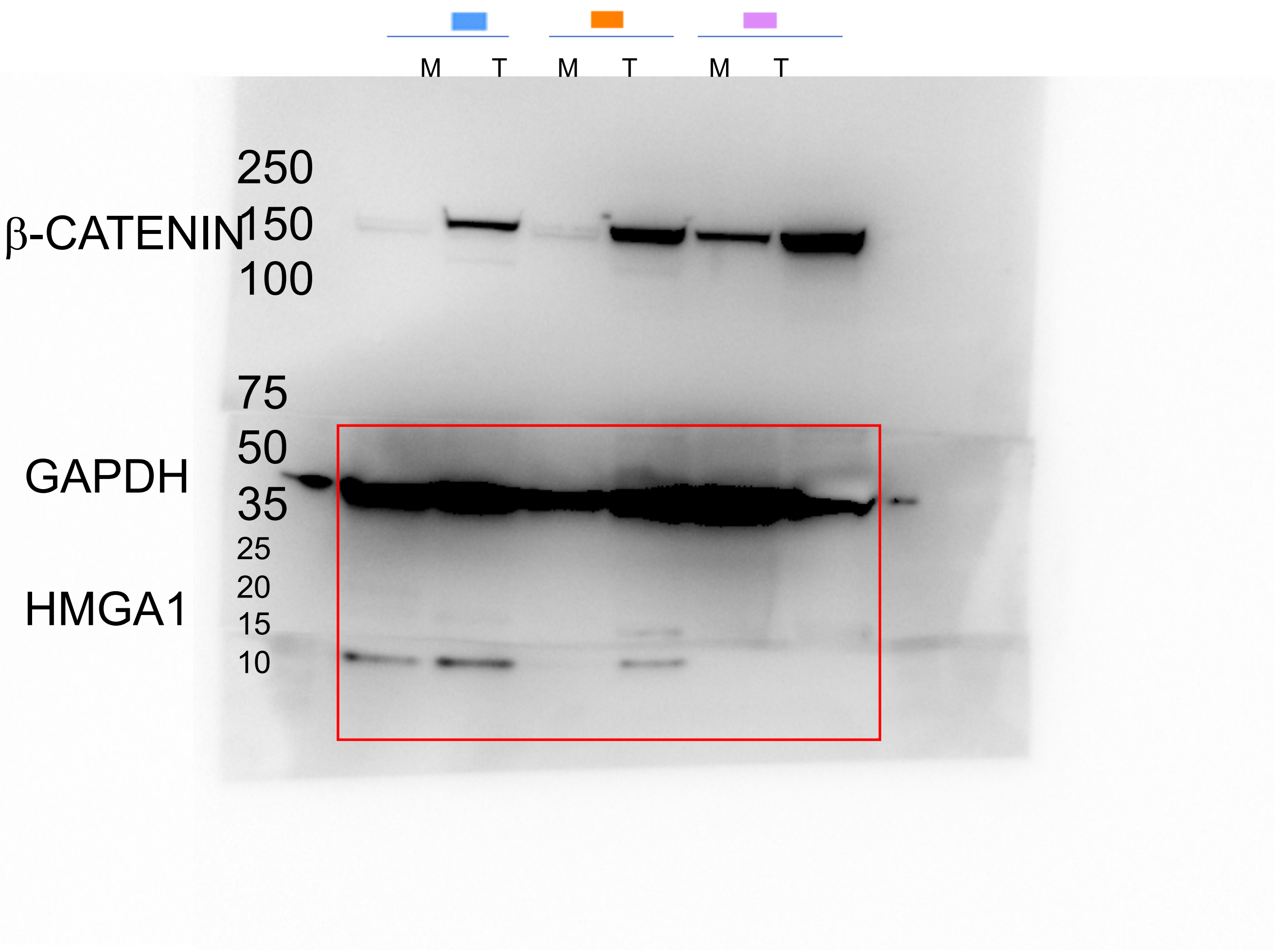

High exposure time

Biological replicate 2 2022-12-01

Full unedited blot for Supplemental Fig. 1B

Biological replicate 3   2022-12-20

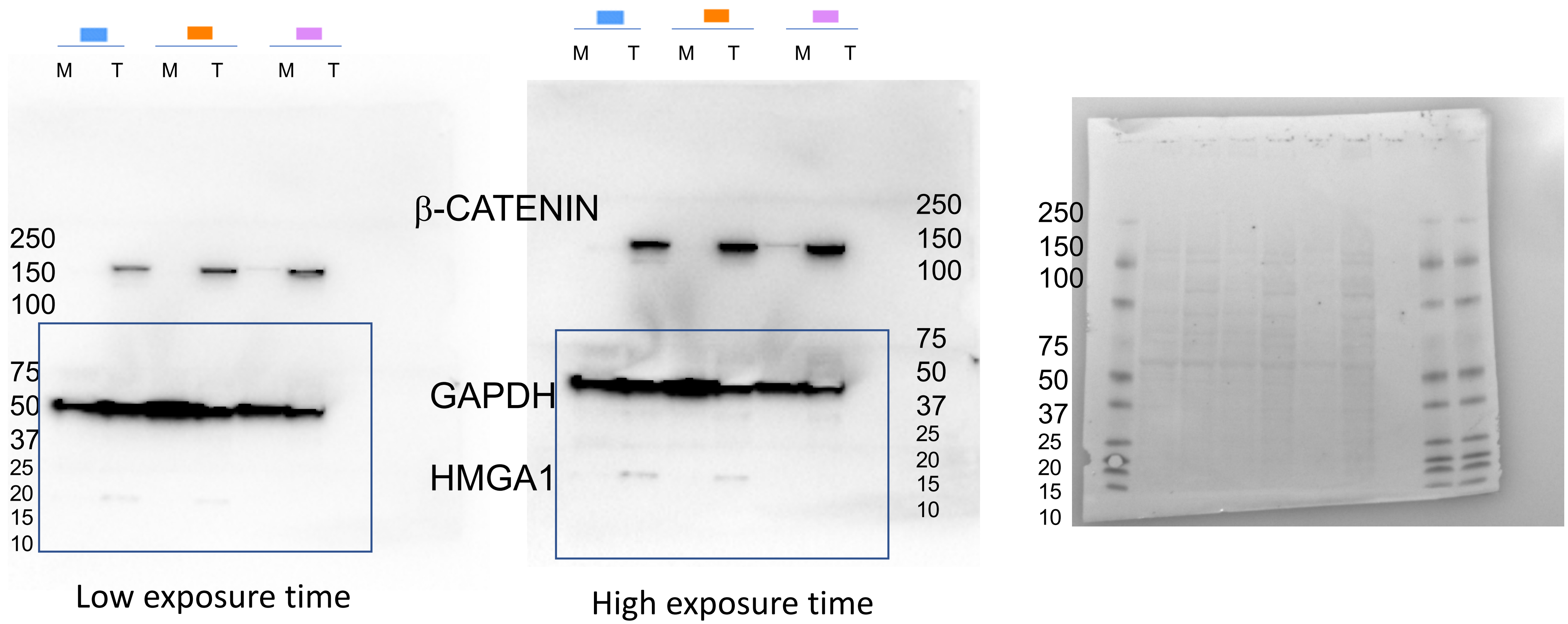

Biological replicate 3   2022-12-20

CDX2P-CreER^{T2} *Apc*$^{fl/fl}$ / *Hmga1*$^{+/+}$
CDX2P-CreER^{T2} *Apc*$^{fl/fl}$ / *Hmga1*$^{+/-}$
CDX2P-CreER^{T2} *Apc*$^{fl/fl}$ / *Hmga1*$^{-/-}$
